# Effects of Combined Exercise Interventions on Cognition in Older Persons: A Systematic Review and Meta-Analysis

**DOI:** 10.3390/healthcare14162600

**Published:** 2026-08-18

**Authors:** Kaan Akalp, José Pedro Ferreira, Carlos Soares, Hicran Besikci, Ana Catarina Bizarro, Maria José Ribeiro, Ana Maria Teixeira

**Affiliations:** 1 Faculty of Sport Science and Physical Education, University of Coimbra, 3040-248 Coimbra, Portugal; 2 Interdisciplinary Center for the Study of Human Performance (CIPER), Faculty of Sport Sciences and Physical Education, University of Coimbra, 3040-248 Coimbra, Portugal; 3 Molecular Physical-Chemistry R&D Unit, Department of Chemistry, University of Coimbra, 3004-535 Coimbra, Portugal; 4 ISCE Research Center (CI-ISCE), Higher Institute of Educational Sciences of Douro, 4560-708 Penafiel, Portugal; 5 Coimbra Institute for Biomedical Imaging and Translational Research (CIBIT), Institute for Nuclear Sciences Applied to Health (ICNAS), University of Coimbra, 3000-548 Coimbra, Portugal; 6 Faculty of Medicine, University of Coimbra, 3000-548 Coimbra, Portugal; 7 RISE-Health, Center for Translational Health and Medical Biotechnology (TBIO), School of Health (ESS), Polytechnic of Porto, 4200-072 Porto, Portugal

**Keywords:** aging, exercise, cognitive function, mild cognitive impairment, cognitive training

## Abstract

**Background/Objectives:** Combining exercise with cognitive training (Combined Intervention) is a strategy against age-related cognitive decline; however, the best exercise type to maximize the neuroprotective effects of combined intervention remains unclear. Therefore, this systematic review and meta-analysis (SRMA) aimed to compare the effects of combined interventions (exercise plus cognitive training) versus exercise-only on cognitive function and blood biomarkers in older persons across different exercise types. **Methods**: PubMed, Scopus, PsycInfo, and Web of Science databases were searched without time restriction. Study quality was evaluated using a modified Downs and Black checklist. Extracted data were analyzed using the random-effects model with Comprehensive Meta-Analysis 4.0 software. **Results:** Twenty-four studies involving 1079 participants (mean age 72.97 ± 5.55 years), with and without mild cognitive impairment (MCI), were included in the present SRMA. Overall, combined interventions demonstrated a small but significant effect size (ES) of 0.335 (95% CI: 0.212 to 0.459; *p* < 0.001) on cognitive function compared to exercise alone. When categorized by exercise type, significant additional cognitive benefits were observed for aerobic (ES = 0.382; *p* < 0.001), multicomponent (ES = 0.261; *p* = 0.005), and resistance exercise (ES = 0.440; *p* = 0.005) combined with cognitive training compared to exercise alone. However, combined interventions showed no significant additional effect on BDNF levels (ES = 0.457; 95% CI: −0.896 to 1.809; *p* = 0.508). **Conclusions**: Incorporating cognitive training into an exercise program showed small but significant additional benefits to overall cognitive function in older persons compared with physical exercise alone. However, the results should be interpreted with caution due to existing limitations.

## 1. Introduction

Aging and a sedentary lifestyle are risk factors for neurocognitive disorders, and with the increase in life expectancy, an increased incidence of neurocognitive disorders is expected [1,2]. Although aging is an unmodifiable risk factor, a sedentary lifestyle can be mitigated through appropriate exercise interventions. Exercise interventions, through their physiological mechanisms, contribute to preserving the structural and functional integrity of the brain and could reduce the conversion rate from Mild Cognitive Impairment (MCI) to dementia [3]. Besides exercise, cognitive training can strengthen neural connections and enhance Brain-Derived Neurotrophic Factor (BDNF) secretion [4]. Therefore, combining exercise and cognitive training could be more effective than exercise alone.

Aging also leads to an elevation in low-grade chronic inflammation and a reduction in neurotrophic factor levels, which eventually cause neurodegeneration and cognitive decline [5,6]. Increased circulating levels of pro-inflammatory cytokines like Interleukin-6 (IL-6), Interleukin-1beta (IL-1β), and tumor necrosis factor alpha (TNF-α) increase blood–brain barrier permeability [7] and can cross the blood–brain barrier through circumventricular organs, endothelial cells, or via blood–brain barrier transporters [8,9]. This increases brain inflammation and causes neurodegeneration, as well as reduced cognitive function [10,11]. Moreover, increased pro-inflammatory cytokine concentrations have been observed in persons with MCI [12]. Exercise can preserve cognition by reducing pro-inflammatory cytokine concentrations and enhancing the release of neurotrophic factors [13].

The effects of exercise on cognition can be influenced by the type of exercise performed [14]. On the one hand, aerobic exercise may affect cognition by increasing BDNF via the peroxisome proliferator-activated receptor-gamma coactivator-1α (PGC-1α)–Fibronectin type III domain-containing protein 5 (FNDC5)–BDNF pathway and by increasing angiogenesis in the brain [15]. On the other hand, resistance exercise preserves cognition by reducing inflammation and increasing insulin-like growth factor-1 (IGF-1) secretion, which contributes to the production of BDNF [16]. Thus, different exercise types activate distinct pathways, which may influence the efficacy of cognitive training and the effects of combined interventions on neuroprotective mechanisms. Indeed, research has demonstrated different effect sizes for distinct types of exercises for cognitive function in cognitively healthy persons and persons with MCI [17,18,19]. To our knowledge, only a small number of studies have investigated the effects of combining exercise with cognitive training (combined intervention) on cognitive function across exercise types [20,21]. In a systematic review, Lauenroth and colleagues concluded that combined cognitive and exercise programs that included both strength and aerobic training were effective in preserving cognitive function. However, the research included mainly multicomponent exercise programs, with a lack of comparability due to different types of control groups [21]. A recent meta-analysis suggested multimodal exercise as the most effective exercise type for combined interventions in improving cognitive function. However, the study included a population with dementia [20], and the effects of exercise can vary between persons with MCI and dementia [22]. Furthermore, negative results from combined intervention compared to physical exercise alone have been reported in persons with or without MCI [23]. Moreover, the design of the cognitive training program may affect cognitive outcomes, depending on which domain is assessed [24,25]. The combined interventions operate within a “Guided-Plasticity-Facilitation” framework. According to this model, physical exercise creates facilitation for neuroplasticity by enhancing secretion of neurotrophic factors (e.g., BDNF), while cognitive training promotes improvements in synaptic connectivity and neural function [26]. Thus, cognitive training can enhance cognitive benefits that originated from exercise alone. Given that exercise types can operate through distinct biochemical mechanisms, as aforementioned, the facilitative effect of exercise may vary across exercise types. To the best of our knowledge, there is no consensus regarding which type of exercise is most effective when combined with cognitive training. Therefore, a comprehensive meta-analytic approach is needed to understand the effects of different types of exercise implemented in cognitive training in older people without dementia.

Although previous studies used a comprehensive approach by combining cognitive scores, they did not evaluate overall cognitive function across exercise types [27,28]. Limited evidence comparing the effects of combined interventions with those of a single type of exercise on cognitive function in older persons highlights the need for new research in this field. Moreover, identifying the most beneficial exercise type to combine with cognitive training is crucial for developing effective strategies that enhance the transfer effect of cognitive training, prevent functional decline, and allow programs to be personalized to optimize brain health in the aging population. Therefore, the main purpose of this SRMA (Systematic Review and Meta-Analysis) was to compare combined interventions (exercise and cognitive training) with exercise alone and to elucidate the effects of combined interventions across different exercise types on overall cognitive function. Our secondary aim was to investigate the effects of combined interventions on blood biomarkers associated with cognitive function.

## 2. Materials and Methods

### 2.1. Search Strategy

A search strategy was developed in accordance with the Preferred Reporting Items for Systematic Reviews and Meta-Analyses (PRISMA) guidelines (Supplementary Table S1) [29] by using the keywords and inclusion criteria defined by the authors, an adapted search strategy for each database, and detailed filters; the number retrieved from each database is presented in Supplementary File (Supplementary Table S2). The keywords used in the search strategy were a combination of Mesh terms and free text words as follow: (“Older Adults” OR “Older Persons” OR “Elderly” OR “Aging” OR “MCI” OR “Mild Cognitive Impairment”) AND (“Dual Task” OR “Multimodal” OR “Cognitive Training” OR “Cognitive Stimulation” OR “Exergame” OR “Exergaming”) AND (“Physical Exercise” OR “Aerobic Exercise” OR “Resistance Exercise” OR “Exercise” OR “Physical Activity”) AND (“Mental health” OR “Cognitive assessment” OR “Memory” OR “Neurogenesis” OR “Neuroplasticity” OR “Brain health” OR “BDNF” OR “IGF-1” OR “IL-6” OR “TNF-alpha” OR “VEGF” OR “Irisin” OR “NFL” OR “Cognition” OR “MMSE” OR “MoCA” OR “Cognitive Function”). The search was conducted in Web of Science, Scopus, PsycInfo and the US National Library of Medicine National Institutes of Health (PubMed) without time restrictions. Advanced research was conducted by using the filters: (a) articles and English for Web of Science; (b) limits used as humans, article and English for Scopus; (c) filters used as clinical study, clinical trial, randomized controlled trial, English, and Humans for PubMed (d) article, exercise, aging, humans filters applied to the PsycInfo database (Supplementary Table S2). Due to the existence of a high number of studies related to cognition, to ensure the capture of specific studies related to our research and to reduce the number of unrelated study designs (e.g., cross-sectional study design, meta-analyses), we used the “clinical trial” and “randomized controlled trial” filters. In order to reduce the number of interventions that did not focus on exercise intervention, we used “exercise” as a filter, and lastly, to reduce the number of studies that used younger populations, we used “aging” as a filter. The search was not performed on reference lists, gray literature, or trial registers. The last search was performed for all databases on 18 May 2026. We included non-randomized studies in this SRMA to compare combined interventions with exercise alone, avoiding clinical data loss and reflecting real-world practice, as randomization may not be achieved due to participants’ preferences even when intended [30,31]. The protocol for this SRMA was registered in INPLASY with the registration number INPLASY202590039.

### 2.2. Eligibility Criteria, Study Selection, and Exercise Classification

The inclusion criteria for the present SRMA were studies: (a) with older persons with or without MCI (>50 years old). This age threshold was selected because age-related cognitive changes, subjective cognitive complaints, and early-onset MCI can be present in individuals aged 50 years and above [32] and this populations are pooled to evaluate overall efficacy of the combined intervention across the pre-dementia cognitive continuum, reflecting how these preventative strategies are implemented across varied cognitive profiles (cognitively healthy individuals, subjective cognitive complaints and MCI) before onset of clinical dementia; (b) evaluating regular or chronic exercise effects; (c) including both combined exercise (with cognitive training) and exercise groups only; (d) with randomized or non-randomized controlled trials; (e) where cognitive function was measured via quantitative assessment tools (e.g., MoCA, MMSE, Stroop Task); (f) with reports published in English language.

The exclusion criteria were studies with: (a) populations with dementia, major depression, Parkinson, multiple sclerosis, schizophrenia; (b) populations with metabolic diseases (e.g., obesity, diabetes); (c) populations with stroke; (d) that assessed supplement consumption effects; (e) with poor quality assessment score according to Downs and Black Checklist; (f) that only included combined (with cognitive training) exercise or only exercise groups; (g) that compared motor learning or level of cognitive training; (h) a different type of exercise in the exercise group from that of the combined intervention; (i) that included biomarkers without evaluating cognitive outcomes; (j) persons with amnestic MCI (this population were excluded because this subtype is associated with Alzheimer disease and presents with distinct memory complaints, thus inclusion of this population could potentially influence the outcome of the study [33]); (k) reports that did not provide appropriate quantitative data for quantitative analysis; (l) post-surgical interventions; (m) population with sarcopenia. The PICOS (Population, Intervention, Comparison, Outcome, and Study Design) framework was used to define the components of the research question [34] which was determined by the authors (K.A., M.R., A.M.T.) as follows:

- Population: Older persons with or without MCI.
- Intervention: Exercise with cognitive training.
- Comparison: Exercise alone (same type of exercise as combined with cognitive training).
- Outcome: Cognitive function measured with quantitative assessment tools.
- Study design: Randomized and non-randomized controlled trials.

Interventions were categorized into four categories: aerobic exercise, resistance exercise, multicomponent exercise, and combined interventions. Aerobic exercises are continuous and rhythmic activities designed to enhance cardiorespiratory fitness [35]. Resistance exercises are activities designed to enhance strength, muscle hypertrophy, and power by using resistance [36]. Multicomponent exercise was categorized as an intervention that combined different types of exercise, such as balance, resistance, and aerobic activities, within the same intervention. Additionally, exercises that incorporated cognitive training, either simultaneously or sequentially, into aerobic, resistance, or multicomponent exercise programs, were categorized as combined interventions.

### 2.3. Data Extraction

**Figure 1:**
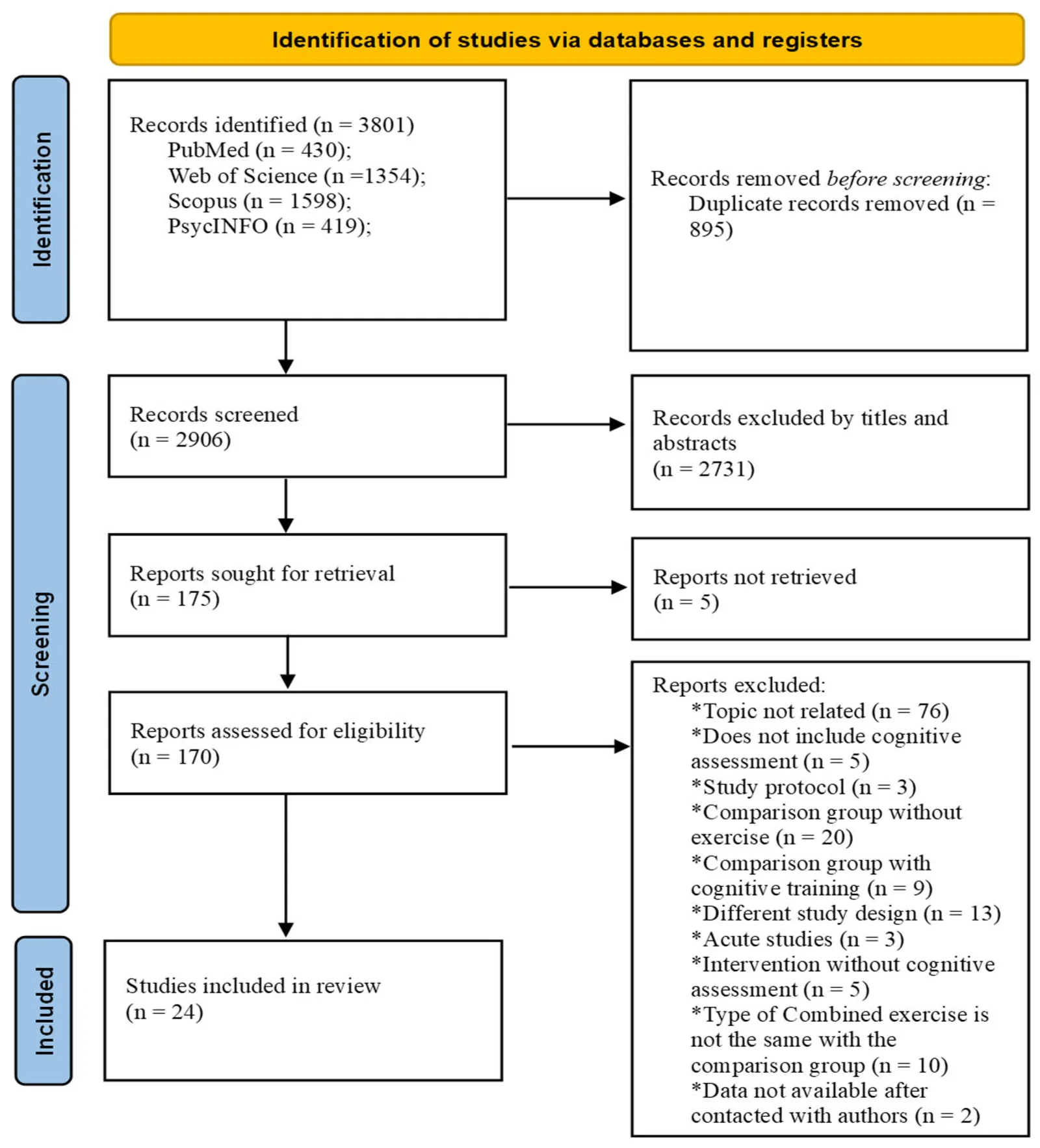
PRISMA Flow Diagram (* indicates exclusion reasons).

The collected data were imported into the EndNote program (Clarivate, Philadelphia, PA, USA, Version 20.2). The imported references were screen to determinate eligibility for the study using the following screening strategy; (a) all duplicates removed; (b) articles were screened by title and abstract, and excluded if they did not include the necessary information about the topic; (c) full text articles that did not meet the inclusion criteria were excluded; (d) methodological quality of the studies was evaluated using the Modified Downs and Black Checklist. Data extraction and screening were performed in accordance with the PRISMA guidelines (Figure 1) [29]. Eligibility criteria were determined by the authors (K.A., M.R., A.M.T.). The elimination by abstract, title, and full-text assessment was performed by two authors (K.A. and H.B.); data extraction was performed by a single author (K.A.); extracted quantitative data were recorded with the data entry interface of Comprehensive Meta-Analysis (CMA) 4.0 (Biostat, Englewood, NJ, USA, version 4.0) software and in a Word document. The extracted data were cross-checked and validated by the third author (J.P.F.) to ensure accuracy and minimize selection bias. In case of disagreements, data were checked from the original source, and any uncertainties were resolved by consulting a third author (A.M.T.). The included studies in this SRMA were reported as follows: authors’ names, study population, age, sample size, exercise type with cognitive task, comparison group, exercise intensity, exercise duration, blood biomarkers, and cognitive outcomes.

### 2.4. Methodological Quality Assessment

The methodological quality of the studies was assessed using a modified version of the Downs and Black Checklist for randomized and non-randomized designs of healthcare interventions [37]. A modification was made to the previous study for scoring item 27, as whether a power calculation was conducted (1) or not (0) [38]. The methodological quality analysis was conducted independently by two authors (K.A. and C.S.). Any disagreements were resolved by consulting a third author (A.M.T.). The checklist assesses five domains: reporting (10 items), external validity (3 items), internal validity-bias (7 items), internal validity-confounding (6 items), and power (1 item). The score ranges from 0 to 28 points, and we used the following cutoffs: poor quality (≤14), fair quality (15–19), good quality (20–25), and excellent quality (26–28) [38].

### 2.5. Statistical Analysis

This meta-analysis was conducted to demonstrate and compare the effects of combined interventions versus exercise-alone programs on overall cognitive function across exercise types, as well as on neuroprotective and inflammatory biomarkers. We contacted three authors whose reported outcomes were not suitable for our analysis. We requested the raw data for pre- and post-means and standard deviations via email. However, only one author provided the requested data. Therefore, we excluded the two other studies.

All statistical calculations were performed using CMA software by choosing the standardized difference in means (SDM) metric (Biostat, Englewood, NJ, USA, version 4.0) [39]. The calculation of effect sizes was performed using data on sample size (N), pre-post combined intervention and exercise group size, means, standard deviations (sd), effect size direction, pre-post correlation (0.5), and score changes. Since the exact pre-post correlation was not reported in the primary studies, a fixed value of r = 0.5 was utilized to calculate the variance of standardized difference in means. This value was chosen because it is reported to be closer to the actual pre-post correlations found in the literature [40]. However, a sensitivity analysis was performed for different correlation coefficients (r = 0.3 and r = 0.7). To ensure the precision of the results, an additional sensitivity analysis was performed by excluding non-randomized trials and the 30 min combined exercise (with cognitive training) group from the multi-arm trial with a single comparison group [41]. After performing sensitivity analyses, the significance of the results was unchanged. However, when we applied a 0.7 pre-post correlation value to the resistance exercise analysis, the effect size slightly varied from a small effect (0.440) to a moderate effect (0.556). Results of the sensitivity analyses are presented in Supplementary Table S3. Assuming that the true effect of exercise and the combined intervention may vary across studies, we used a random-effects model that incorporates sampling error and between-study variance [42,43]. For studies that used tests in which lower scores indicate better cognitive function, we reversed the effect size direction from negative to positive. If the studies reported more than one outcome, the multiple outcomes were combined by using the mean of effect sizes with between-study correlation as 1; this conservative approach underestimates the precision of the summary effect and overestimates variance, which reduces the risk of type1 error, and effect sizes were reported as a single effect size to avoid double counting participants [44,45]. Further description of the cognitive tests included can be found in Supplementary Table S4. The effect sizes were categorized as trivial (d ≤ 0.20), small (0.21 ≤ d ≤ 0.50), moderate (0.51 ≤ d ≤ 0.79), and large (d ≥ 0.80) [46].

The heterogeneity between studies was assessed using the intercept value, confidence interval, degrees of freedom (df), and two-tailed *p*-value. To evaluate the null hypothesis, Cochran’s Q statistic was used, and the results were reported as the z-value and corresponding *p*-value from the test of the null hypothesis. To assess the distribution of effect sizes, the Q-value (Q), df, I-squared (I^2^), and the *p*-value from the heterogeneity test were used [47]. The variation in true effect sizes among studies was evaluated using the Tau-squared (τ^2^), whilst the standard deviation of the true effect magnitudes was reported using the Tau (τ) value [43].

The publication bias was calculated using CMA software (Biostat, Englewood, NJ, USA, version 4.0) [39] using Egger’s regression test and visually assessed by creating a funnel plot of standard errors and standard differences in means [43].

## 3. Results

### 3.1. Data Search and Selection

The search was conducted across four databases, and 3801 studies were identified (Figure 1). Although no initial time limit was applied to ensure the inclusion of all relevant studies, the final search date (18 May 2026) allowed us to include the most recent publications related to combined intervention and cognitive function. During the identification process, 895 duplicates were removed. A total of 2906 studies were screened, and 2731 studies were excluded based on title and abstract analyses. During the eligibility evaluation, five reports were unavailable, and 146 studies were excluded for not meeting the inclusion criteria of this SRMA. Additionally, despite their scientific relevance, we excluded two studies [48,49] because we were unable to obtain the missing data, even after requesting them from the authors via email. However, we included two non-randomized studies [50,51]. Although one study reported a raffle for assigning participants, the authors included lack of randomization as a limitation. Therefore, we classified this study as a non-randomized trial [50]. The 24 studies that met our eligibility criteria were assessed for methodological quality using the Downs and Black Checklist (Supplementary Table S3) [37,38].

### 3.2. Characteristics of the Studies and Participants

The summary of the study characteristics in terms of study name, study population, age, and sample size, exercise type with cognitive training, comparison groups, exercise intensity, duration, biomarkers, and cognitive outcomes is presented in Table 1. Additionally, Supplementary Table S5 presents study quality scores according to the modified version of the Downs and Black Checklist. This SRMA included five studies of fair quality (scored 15–19), 18 studies of good quality (scored 20–25), and one study of excellent quality (scored > 25). Twenty-four studies with a total of 1079 participants, with an average age of 72.97 ± 5.55 years, including participants with and without MCI.

### 3.3. Comparison of Global Effects of Exercise and Combined Intervention on Cognitive Function

**Figure 2:**
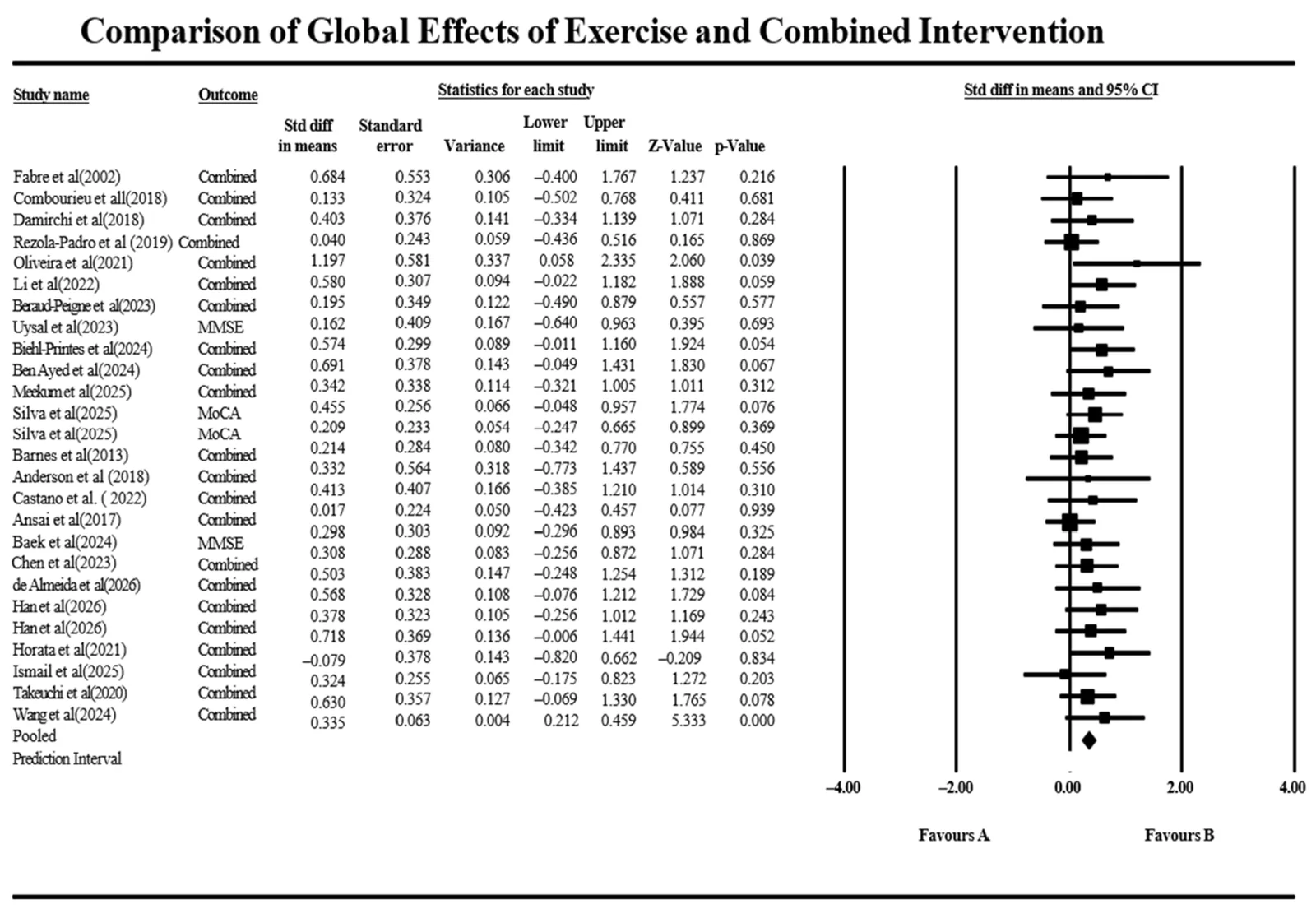
Forest plot of comparison of overall effects of exercise and combined intervention on cognitive function (Silva et al., 2025 [71] indicate the same study with two different types of exercise; Han et al., 2026 [41] indicate the same study with two different combined interventions) [41,50,51,52,53,54,55,56,57,58,59,60,61,62,63,64,65,66,67,68,69,70,71,72].

Our results indicate an ES of 0.335 (standardized difference in means) between the exercise and the exercise with cognitive training groups using the random-effects model. This result demonstrates that participants who were involved in the combined intervention groups had 0.335 higher average scores in cognitive function performance than the exercise-only groups (Figure 2). The CI for the ES is 0.212 to 0.459 (95% CI). The Z-value from the test of the null hypothesis is 5.333 with *p* < 0.001. This result indicates that combined interventions have significantly better effects on cognitive function performance, in comparison with exercise alone in older persons with or without MCI. The Q-value is 13.435 with 25 degrees of freedom (df). Due to a Q-value less than the df, the variance of true effects is estimated as zero and indices of heterogeneity (I^2^, τ^2^, and τ) are set to zero. Estimation of τ^2^ as zero indicates that all studies share a common effect size; therefore, we are not able to report a prediction interval. The results obtained from Egger’s regression show an intercept of 1.625 with 0.49 to 2.75 (95% CI), τ = 2.97, df = 24, and *p* = 0.007 (2-tailed). Visual Inspection of funnel plots shows asymmetry originating from the bottom of the funnel plot, indicating that smaller studies with negative or null results are missing (Supplementary Figure S1) [44,73,74,75,76,77,78,79,80,81,82]. Additionally, analysis of the simultaneous delivery method revealed an ES of 0.331 (standardized difference in means) with 0.145 to 0.557 (95% CI) between exercise and combined intervention groups in the random-effects model. The Z-value is 4.565 with *p* < 0.001 (Supplementary Figure S2a). Q-value is 12.117 with 19 df. Therefore, I^2^, τ^2^, and τ are set to zero. Egger’s regression intercept is 1.831 with 0.298 to 3.363 (95% CI), τ = 2.510, df = 18, and *p* = 0.02 (2-tailed). This result suggests publication bias or small-effect studies within the meta-analysis (Supplementary Figure S2b). The analysis of the sequential delivery method revealed an ES of 0.347 (standardized difference in means) with 0.101 to 0.593 (95% CI) between the exercise and the combined intervention group in the random-effects model. The Z-value is 2.760 with *p* = 0.006 (Supplementary Figure S3a). Q-value is 1.306 with 5 df. Therefore, I^2^, τ^2^, and τ are set to zero. Egger’s regression intercept is 1.268 with −0.460 to 2.998 (95% CI), τ = 2.036, df = 4, and *p* = 0.11 (2-tailed) (Supplementary Figure S3b). These results indicate that both sequential and simultaneous delivery methods have a small but significant effect on overall cognitive function compared to the exercise-only group, without showing statistical evidence for publication bias.

### 3.4. Comparison of Effects of Exercise with Combined Intervention on BDNF

**Figure 3:**
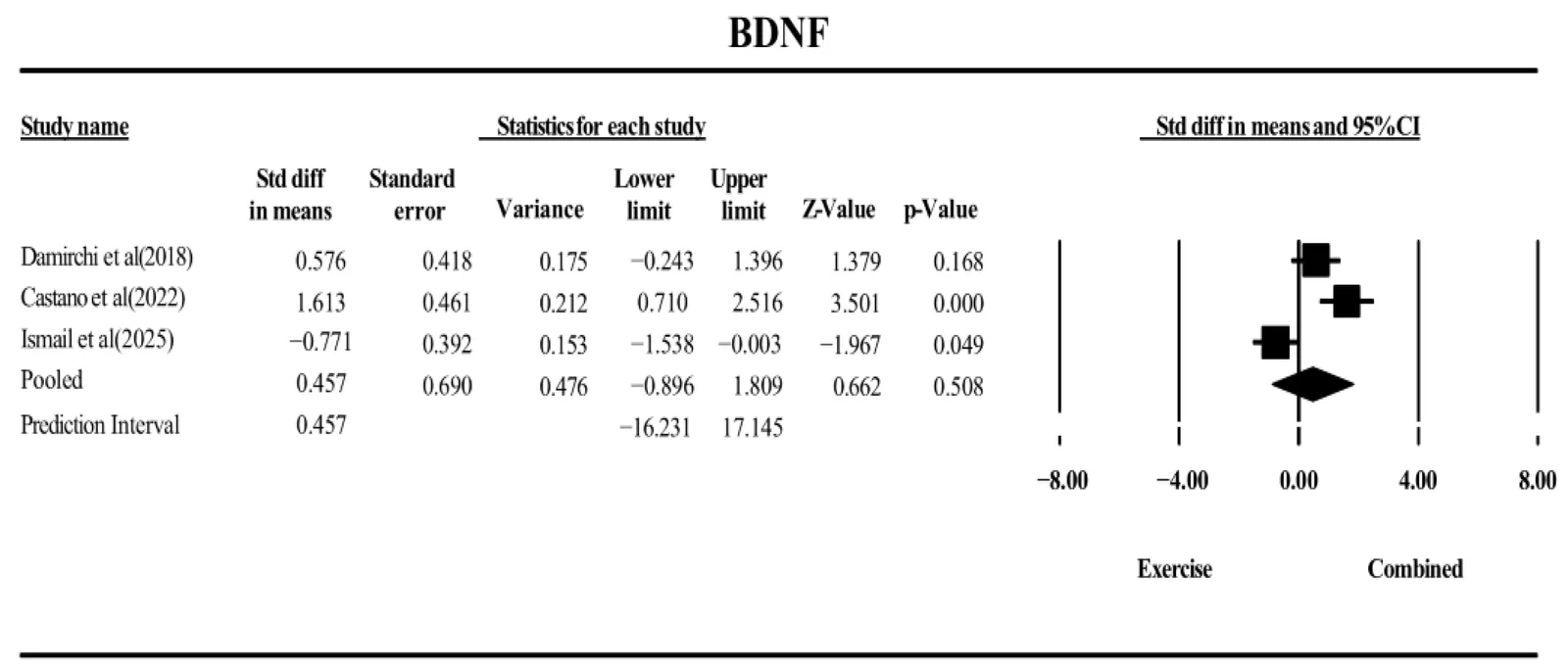
Forest plot of comparison of the overall effects of exercise and combined intervention on BDNF [56,61,69].

Our results indicate an ES of 0.457 (standardized difference in means) between the exercise and the exercise with cognitive training groups using the random-effects model. This result demonstrates that participants who were involved in the combined intervention groups had a 0.457 higher increment in average BDNF levels than the exercise-only groups (Figure 3). The CI for the ES is −0.896 to 1.809 (95% CI). The Z-value from the test of the null hypothesis is 0.662 with *p* = 0.508. This result indicates that combined interventions have no significant effect on BDNF, in comparison with exercise alone, in older persons with or without MCI. The Q-value is 15.990 with 2 degrees of freedom (df) and *p* < 0.001. The I^2^ statistic of 87.49 indicates that 88% of the observed variance reflects the variance of true effects. τ^2^ is 1.249, and τ is 1.117 in d units. Assuming a normal distribution of true effects, the prediction interval is −16.231 to 17.145, indicating that the true effect size in 95% of comparable populations falls within this interval. The results obtained from Egger’s regression show an intercept of 34.197 with −60.71 to 129.11 (95% CI), τ = 4.57, df = 1, and *p* = 0.137 (2-tailed). These results indicate that there is no statistical evidence of publication bias (Supplementary Figure S4) [44,73,74,75,76,77,78,79,80,81,82].

### 3.5. Comparison of Effects of Aerobic Exercise with Combined Intervention on Cognitive Function

**Figure 4:**
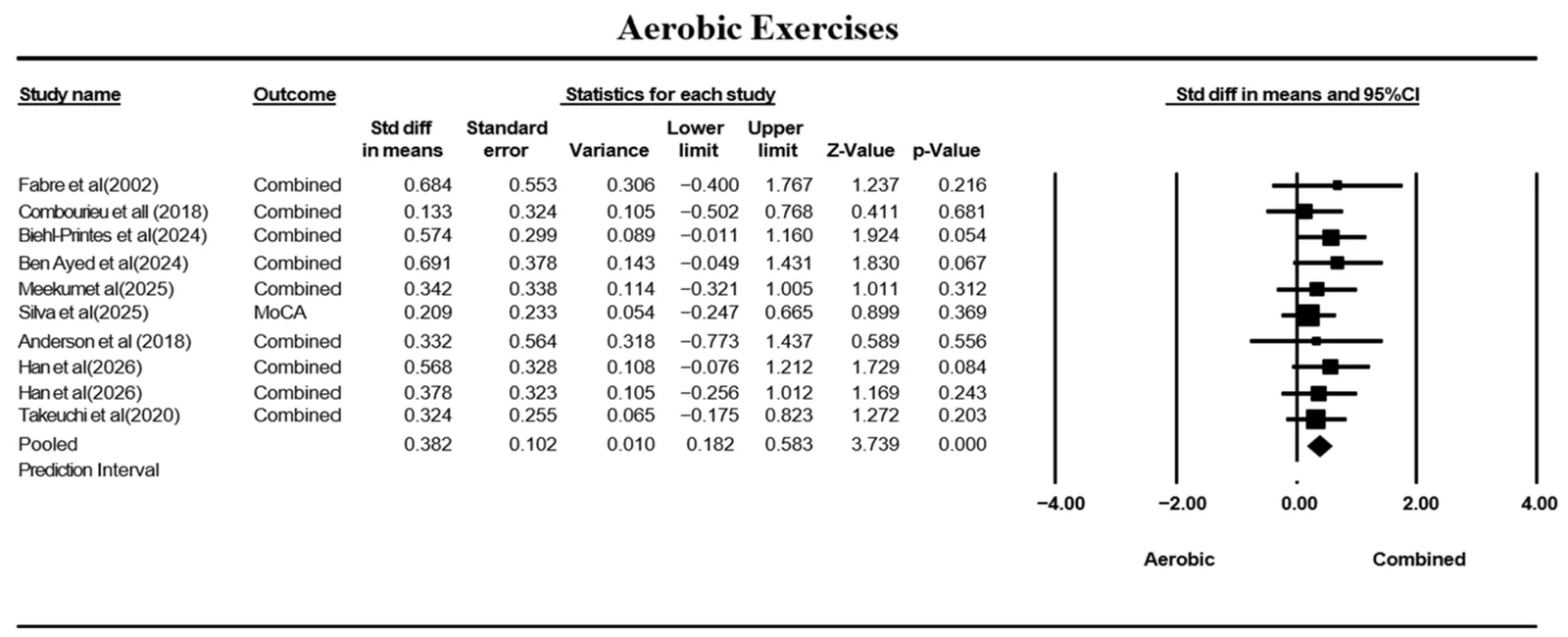
Forest plot of the comparison of the effects of aerobic exercise and combined aerobic exercise with cognitive training. (Han et al., 2026 [41] indicate the same study with two different combined interventions) [41,52,54,55,58,65,67,70,71].

The meta-analysis results indicate an ES of 0.382 (standardized difference in means) between the aerobic exercise and combined aerobic exercise with cognitive training groups in the random-effects model. Participants who were involved in aerobic exercise with cognitive training had a 0.382 higher average score in cognitive function than the aerobic exercise-only group (Figure 4). The CI (95%) for the ES is 0.182 to 0.583. The Z-value is 3.739 with *p* < 0.001. This result indicates that combining aerobic exercise with cognitive training interventions has significantly better effects on cognitive function performance compared to aerobic exercise alone in older persons with or without MCI. Q-value is 2.920 with 9 df; therefore, the variance of true effects was estimated as zero, and indices of heterogeneity (I^2^, τ^2^, and τ) were set to zero. Because τ^2^ was estimated as zero, we were unable to report a prediction interval. The Egger regression demonstrates an intercept of 1.011 with −0.587 to 2.610 (95% CI), τ = 1.458, df = 8, and *p* = 0.182 (2-tailed). These results indicate that there is no statistical evidence for publication bias (Supplementary Figure S5) [44,73,74,75,76,77,78,79,80,81,82].

### 3.6. Comparison of Effects of Multicomponent Exercise with Combined Intervention on Cognitive Function

**Figure 5:**
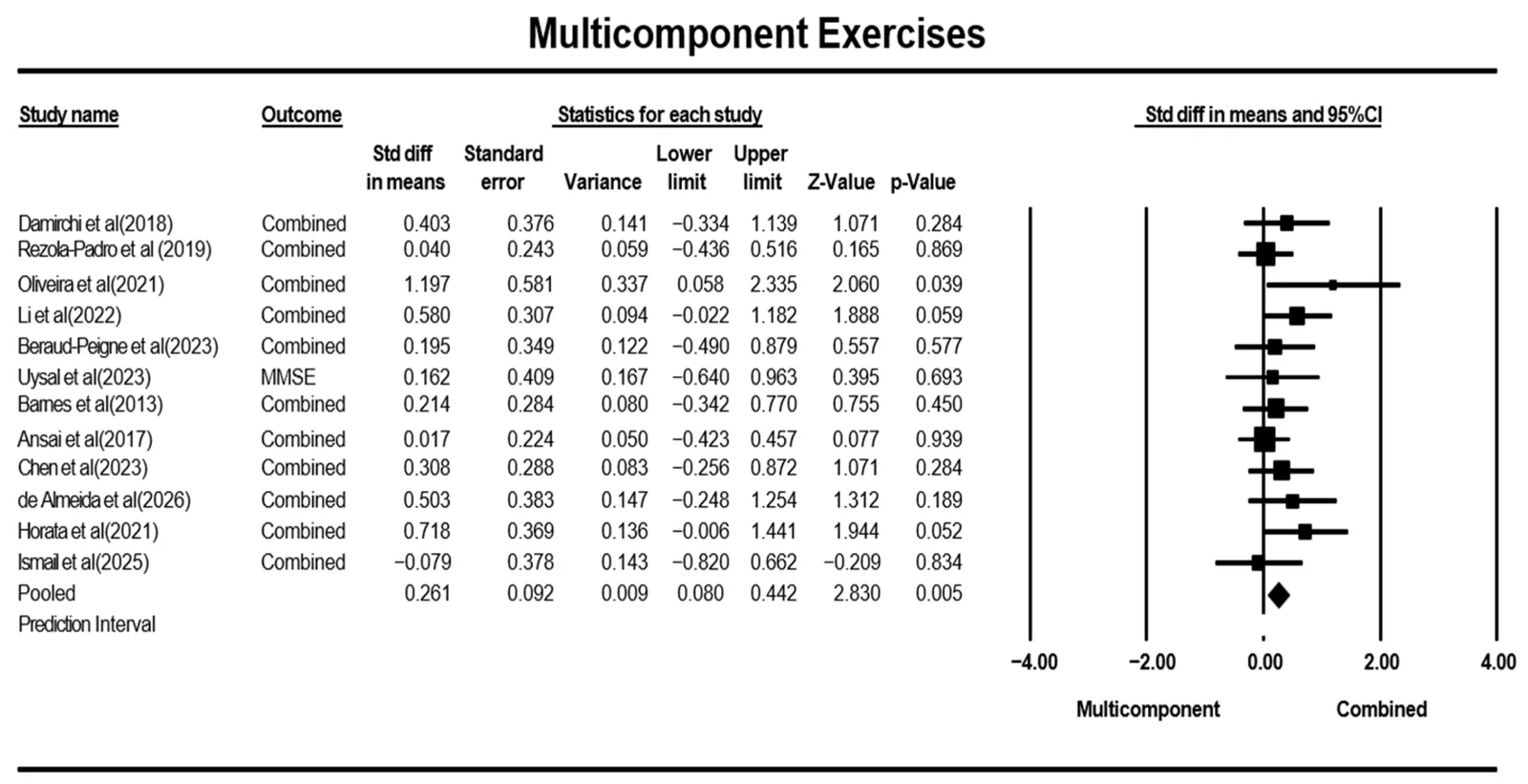
Forest plot of comparison of the effects of multicomponent exercise and combined multicomponent exercise with cognitive training. [50,51,53,56,57,59,60,62,63,64,69,72].

The random-effects model revealed an ES of 0.261 (standardized difference in mean) between the multicomponent exercise groups and the multicomponent exercise combined with cognitive training groups. This result indicates that participants in the multicomponent exercise with cognitive training group had, on average, 0.261 higher cognitive function scores than those in the multicomponent exercise-only group (Figure 5). The CI (95%) for ES is 0.080 to 0.442. The Z-value is 2.830, with a *p*-value of 0.005. This result indicates that multicomponent exercise with cognitive training has significantly better effects on cognitive function performance than multicomponent exercise alone in older persons with or without MCI. The Q-value is 8.710 with 11 df; therefore, the variance of true effects is estimated as zero, and indices of heterogeneity (I^2^, τ^2^, and τ) are set as zero. Because τ^2^ is estimated as zero, we cannot report a prediction interval. Egger’s regression demonstrates an intercept of 2.387 with 0.433 to 4.340 (95% CI), τ = 2.722, df = 10, and *p* = 0.021 (2-tailed). Visual Inspection of the funnel plot shows asymmetry originating from the bottom of the funnel plot, indicating that smaller studies with negative or null results are missing (Supplementary Figure S6) [44,73,74,75,76,77,78,79,80,81,82].

### 3.7. Comparison of Effects of Resistance Exercise with Combined Intervention on Cognitive Function

**Figure 6:**
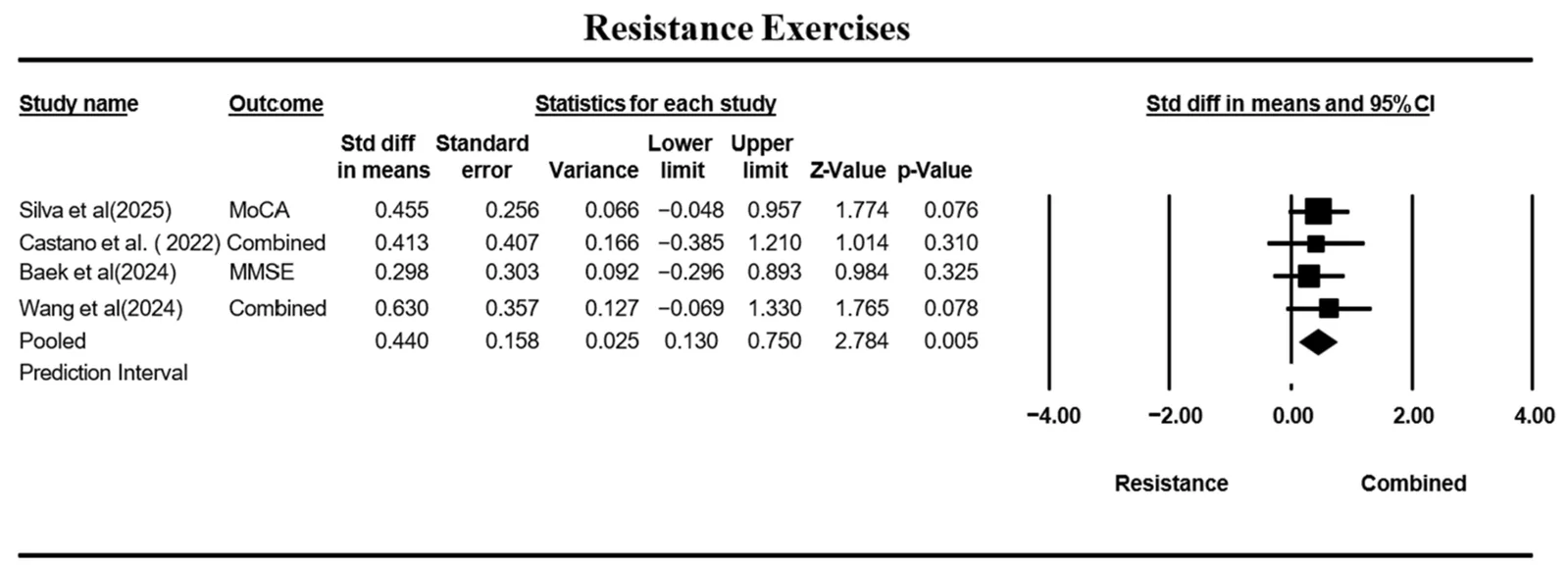
Forest plot of comparison of the effects of resistance exercise and combined intervention on cognitive function in older persons [61,66,68,71].

The random-effects model revealed an ES of 0.440 (standardized difference in means) between the resistance exercise group and the resistance exercise combined with cognitive training group. This result indicates that participants who were involved in combined resistance exercise and cognitive training had, on average, 0.440 higher cognitive function scores than the resistance exercise-only group (Figure 6). The CI (95%) for ES is 0.130 to 0.750. The Z-value is 2.784, with a *p*-value of 0.005. The Q-value is 0.510 with 3 df. Therefore, the variance of true effects is estimated as zero, and indices of heterogeneity (I^2^, τ^2^, and τ) are set to zero. Because τ^2^ is estimated as zero, we cannot report a prediction interval. Egger’s regression demonstrates an intercept of 0.445 with −5.724 to 6.614 (95% CI), τ = 0.310, df = 2, and *p* = 0.785 (2-tailed). These results indicate that there is no statistical evidence for publication bias (Supplementary Figure S7) [44,73,74,75,76,77,78,79,80,81,82].

## 4. Discussion

The primary aim of this SRMA was to compare the effects of exercise alone and combined interventions on cognitive function in older persons with or without MCI. Our SRMA revealed a significantly small effect of the combined interventions over the exercise-only interventions on cognitive function (ES = 0.335; 0.212 to 0.459 (95% CI); *p* < 0.001) and a non-significant small effect on BDNF (ES = 0.457; −0.896 to 1.809 (95% CI); *p* = 0.508) in older persons with or without MCI. Additionally, our results demonstrated similar effects on the cognitive function of older persons with or without MCI, when we classified the combined interventions as aerobic exercise with cognitive training (ES = 0.382; 0.182 to 0.583 (95% CI); *p* < 0.001), multicomponent exercise with cognitive training (ES = 0.261; 0.080 to 0.442 (95% CI); *p* = 0.005) and resistance exercise with cognitive training (ES = 0.440; 0.130 to 0.750 (95% CI); *p* = 0.005) in comparison between the same type of exercise that was applied with the cognitive training. However, heterogeneity is observed in the quantitative analysis of BDNF.

### 4.1. Comparison of Overall Effects of Exercise with Combined Intervention

Our results demonstrate that exercise combined with cognitive training offers additional benefits over exercise alone in improving cognitive function in older persons with or without MCI. Due to different mechanisms at play depending on the type of exercise performed, which could enhance these effects, we only included studies that applied the same type of exercise in both the combined intervention and the exercise-only group. Our results are in line with a previously published meta-analysis [27].

Exercise is considered a stressor that creates adaptation in the neuroendocrine system [83]. Regular exercise decreases pro-inflammatory cytokines, elevates basal levels of anti-inflammatory cytokines [84], and growth factors such as IGF-1, BDNF, and VEGF [85]. Changes in the aforementioned exerkines may exert neuroprotective effects and slow down cognitive decline with aging [86]. Additionally, repeated cognitive stimulation strengthens synaptic connections, helps form new synapses [87], and improves cognitive function [88]. Moreover, cognitive training may reduce stress reactivity by increasing cognitive control [89], and Pesce and colleagues demonstrated that a memory training program reduced cortisol and pro-inflammatory cytokine concentrations (e.g., IL-6, IL-1β, IL-18). It is possible that cognitive training may also exert an inhibitory role on the hypothalamic-pituitary axis [90] and protect against neurodegeneration [91]. Thus, plausible explanations for our results suggest that the combination of exercise and cognitive training may exert synergistic effects through the aforementioned mechanisms, leading to better cognitive performance compared to exercise alone. In the pathophysiology of mood and cognitive disorders, it is well documented that modulating neuroendocrine targets (e.g., via pharmacological treatments) can subsequently influence neurotrophic pathways to provide neuroprotection [92,93]. Similar to these effects, non-pharmacological interventions-such as combined exercises (with cognitive training) appear to harness similar pathways. The neuroprotection provided by combined exercises, potentially through the upregulation of BDNF, may offer a safer alternative or complementary approach to pharmacological treatments for mitigating the neuroendocrine dysregulation commonly seen in cognitive decline, aging, and mood disorders [94,95]. Damirchi and colleagues demonstrated a significant increment in BDNF and IRISIN levels, as well as better working memory performance after 8 weeks of intervention in the combined intervention group, without any significant change in the multicomponent exercise-only group in older women with MCI [56]. However, in the study by Ismail and Yusof, no improvement was observed in BDNF levels after both 8 weeks of exercise and exergame intervention in the persons with cognitive impairment [69]. Moreover, Castaño and colleagues showed significant improvement in BDNF along with semantic verbal fluency (SVF) and phonological verbal fluency (PVF) scores when cognitively healthy participants performed resistance exercise with cognitive training in comparison to the resistance exercise only group for 16 weeks [61]. Additionally, Anderson-Hanley and colleagues demonstrated a partial correlation between BDNF and the number of exercise sessions, indicating that higher BDNF levels are associated with a higher frequency of aerobic exercise. However, their sample size was limited [54]. Thus, the effects of exercise on BDNF potentially change depending on exercise frequency. In our previous meta-analysis, we demonstrated significant effects of exercise on TNF-α levels and a non-significant effect on BDNF levels in comparison with control groups in older persons with MCI [96]. Thus, a combination of cognitive training with exercise may engage complementary biochemical mechanisms that could improve cognitive function. Due to insufficient data, we were unable to conduct a meta-analysis for inflammatory and other neuroprotective blood biomarkers. Given the high heterogeneity and the small number of studies, our results regarding BDNF should be interpreted cautiously. Furthermore, it should be noted that a wide range of prediction intervals is reported in the present SRMA. This variation may have originated from the different assessment methods used, which had varying detection rates. For instance, Damirchi and colleagues utilized an ELISA kit with a detection range of 7.8 to 500 pg/mL for serum samples [56]. In contrast, Ismail and colleagues used a different detection range and reported serum concentrations in ng/mL [69], which likely contributed to the wider range of prediction intervals observed. It should be noted that prediction intervals are founded on the normal distribution of effects, and that at least ten studies included them [39,79]. Additionally, larger effects of exercise on BDNF have been reported in exercise studies with 3–4 sessions per week at moderate to vigorous intensity with a minimum of 12 weeks, regardless of exercise type [97]. In the present SRMA, one study applied 16 weeks of intervention [61], and all of the studies that measured BDNF, besides cognitive function, have applied a moderate intensity level. Thus, this may influence the results in the present SRMA. Further studies that compare the effects of exercise and combined interventions on cognitive function should assess changes in inflammatory and neuroprotective blood biomarkers to clarify the mechanisms underlying neuroprotection.

### 4.2. Comparison of Effects of Combined Aerobic and Cognitive Exercise Versus Aerobic Exercise Alone

In our study, a total of nine studies investigated the effects of a combination of aerobic and cognitive exercise with aerobic exercise only [41,52,54,55,58,65,67,70,71]. Beneficial effects of exercise are dependent on frequency, duration, intensity, and type of exercise [98]. Moderate intensity aerobic exercise is a commonly used method in the studies included in our SRMA. Fabre and colleagues applied interventions at personalized exercise intensity by using the ventilatory threshold [52]. Exercise intensity at ventilatory threshold is considered a moderate level [52]. Although Fabre and colleagues demonstrated that both aerobic exercise and aerobic exercise with cognitive training improved memory quotient, paired-associate learning, and logical memory-immediate recall to a similar significance level, the difference in memory quotient in the combined intervention group was higher in comparison to the aerobic exercise group [52]. Similarly, Anderson-Henley and colleagues found that both types of intervention improved executive function without significant differences after 6 months of intervention [54]. Combourieu Donnezan and colleagues demonstrated additional benefits of a combined intervention on executive performance after 12-week training with moderate intensity. Although both aerobic and combined intervention groups improved similarly in the matrix reasoning test, only the combined group showed significant improvement in the digit span forward and backward tests [55]. On the other hand, Meekum and colleagues did not report additional improvements on the digit span forward and backward tests after the combined intervention. However, they found significant improvement in the Trail Making Test B-A in the combined intervention group when adjusted for age and education [70]. Biehl-Printes and colleagues reported significant improvements in processing speed, attention, and visuospatial skills in both the orienteering (combined intervention) and hiking groups. However, improvement in cognitive function was greater in the orienteering group. Moreover, significant improvement was only observed in the orienteering group for memory skills [67]. Those observations indicate that the benefits of combined intervention may be associated with the type and intensity of the cognitive stimulation when combined with aerobic exercise.

### 4.3. Comparison of Effects of Combined Multicomponent and Cognitive Exercise Versus Multicomponent Exercise Alone

Our results indicated that combined multicomponent programs have a significant effect on overall cognitive function in comparison to a multicomponent program without cognitive training in older persons. We included a total of 12 studies that compared a combination of multicomponent exercise and cognitive training with multicomponent exercise only [50,51,53,56,57,59,60,62,63,64,69,72]. Barnes and colleagues reported a significant difference between groups that participated in a mental intervention and those that did not, regarding visuospatial functions (processing speed, divided and selective attention) in older persons with cognitive complaints [53]. Damirchi and colleagues demonstrated the favorable effects of a combination of multicomponent exercise and cognitive training on working memory performance compared with exercise only [56]. However, they did not observe significant differences in processing speed, reaction time, and error numbers in older women with MCI [56]. On the other hand, Rezola-Pardo and colleagues did not observe additional benefits of combining multicomponent exercise with cognitive training on cognitive function [57]. Additionally, Uysal and colleagues observed similar findings on MMSE when comparing the aerobic exercise plus lower-limb strengthening group with the group that additionally performed dual-task exercise [64]. In contrast, an exergame intervention showed greater improvement in delayed maze time than the multicomponent exercise group, which performed the same movements without cognitive stimulation [60]. The inconsistent findings in the literature regarding the combination of multicomponent exercise and cognitive training may stem from the nature of multicomponent exercise. Multicomponent exercises consist of a variety of exercise types and modalities. Thus, the proportion of aerobic or resistance exercise components may vary across studies. The physiological stimuli involved in multicomponent exercise may overlap, affecting neuroplasticity in various ways. Aerobic exercise can enhance BDNF through the PGC-1α pathway, while resistance exercise can enhance IGF-1 to modulate neuroplasticity [15,16]. Since these adaptations are not strictly exclusive, the magnitude of cognitive enhancement or the release of neurotrophic factors can vary depending on how these types of exercise (aerobic and resistance) are structured [16]. Additionally, Vanegas-Sanabria and colleagues indicate that positive effects of multicomponent exercises on global cognitive function depend on the inclusion of aerobic exercise [99]. On the other hand, Loprinzi and colleagues suggested that the additive effect of multicomponent exercise may originate from resistance exercise [16]. Consequently, the nature of the multicomponent interventions makes it challenging to isolate the specific factors that promote cognitive improvement. Moreover, including different modalities may enhance cognitive enrichment, which can have effects similar to cognitive training [100].

### 4.4. Comparison of Effects of Resistance and Cognitive Exercise Versus Resistance Exercise Alone

Our SRMA results indicate a small but significant effect of resistance exercise with cognitive training on cognitive function compared to resistance exercise alone. Specifically, we analyzed four studies that investigated this comparison [61,66,68,71]. In recent SRMA studies, moderate-intensity resistance exercises were applied, and one study utilized a sequential delivery method. Over a 12-week period, Silva and colleagues observed that a sequentially combined strength and cognitive training intervention resulted in similar improvements in MoCA scores when compared to strength training alone in persons with cognitive impairment [71]. Conversely, Baek and colleagues reported that a 6-week dual-task resistance exercise program yielded greater improvements in MMSE scores compared to resistance exercise alone in older persons with cognitive impairment [66]. Similarly, Wang and colleagues demonstrated significant improvements in Trail Making Test and Digit Symbol Substitution Test scores for the simultaneous resistance and cognitive training group compared to the isolated resistance exercise group over an 8-week period [68]. Furthermore, Castano and colleagues reported improvements in SVF and PVF for the simultaneous resistance exercise group over a 16-week period compared to the resistance-only group [61]. Exercise plays a crucial role in the “guided plasticity facilitation” framework during simultaneously combined exercise and cognitive training; thus, performing exercise sequentially can reduce the facilitation effect of exercise, which may attenuate the cognitive adaptation [26,101]. Indeed, one study showed that simultaneous exercises improved cognitive function while sequential tasks yielded inconsistent results [102]. Although we observed similar benefits of sequentially and simultaneously combined exercise, we are unable to perform this analysis for resistance exercise due to the scarcity of studies in the recent SRMA. Furthermore, results on the delivery method of combined interventions showed comparable, small effect sizes, indicating that both sequential and simultaneous delivery methods are effective and promote additional benefits in comparison to exercise alone. However, the guided effects of cognitive training may depend on the interval time between exercise and cognitive training. In practice, when applying a sequential-delivery-method intervention design, caution should be taken [101]. To comprehensively understand synergistic effects, further studies are needed to compare sequential and simultaneous delivery methods across exercise types and their effects on cognitive functions.

## 5. Limitations

The results of our SRMA should be analyzed considering some limitations. The first limitation is that we did not separately analyze healthy persons and persons with MCI. Studies that included healthy older persons with subjective cognitive impairment and persons with cognitive complaints may have had the presence of undiagnosed MCI. Although exercise is beneficial in both MCI and cognitively healthy populations, exercise yields more pronounced effects on cognition when it is impaired [103,104]. The inclusion of both MCI and cognitively healthy populations can potentially cause different effects on cognitive outcomes. In order to align with our primary research aims, our strict search strategy excluded studies that reported comparison groups as only passive or only cognitive training. Thus, recent meta-analyses may not include all available literature regarding the association between biochemical biomarkers and cognitive outcomes. However, our secondary purpose was to directly compare the effects of cognitive and physical training with matched exercise types to elucidate the biochemical mechanisms associated with exercise types and additional benefits of cognitive training. Another limitation is that we used composite scores; therefore, we cannot draw conclusions regarding a specific cognitive domain or skill. However, our purpose was to demonstrate the additional benefits of combined intervention, according to exercise types, on overall cognitive function, rather than on specific cognitive domains. Moreover, the design of cognitive training (type and targeted domain) may influence the results of cognitive tests [24] which evaluate different cognitive domains rather than just the trained ones. Additionally, it is important to note that the inclusion of only cognitively healthy, impaired, and MCI populations limits the generalizability of the present results. Furthermore, when we performed a sensitivity analysis by adjusting pre-post correlation to 0.7, the effect size of resistance exercise analysis shifted from small to moderate. Given the small number of studies included in the present SRMA, we used a conservative pre-post correlation of 0.5 [40].

## 6. Practical Implementation

The findings from our SRMA emphasize the importance of incorporating cognitive training, regardless of the form, alongside either aerobic, resistance, or multicomponent exercise to enhance the cognitive benefits of physical activity. Our results indicate small but significant additional benefits of combined interventions over physical exercise alone. Practically, it allows practitioners to tailor the physical exercise component to the individual’s physical capacity and personal preferences without compromising cognitive outcomes. This flexibility is crucial for improving participants’ adherence to exercise programs [105]. Thus, community fitness programs targeting older adults should integrate cognitive stimulation into physical exercise programs. Cognitive training’s effect on biochemical markers has been reported in the literature [90]. Although we are not able to provide comprehensive quantitative analysis regarding inflammatory biochemical markers, it is crucial to identify the benefits of combined interventions (including cognitive training) compared with exercise alone to elucidate the synergistic effects of those interventions. The studies included in our SRMA primarily focused on low- to moderate-intensity exercises. Exercise intensity is a crucial factor that could potentially affect the cognitive outcomes. Although research indicates that cognitive function may be negatively impacted after acute high-intensity exercise [106], the long-term effects of high-intensity training remain unclear. Given that ACSM recommends at least 75 min of vigorous-intensity exercise per week [107], future studies should investigate long-term cognitive effects of high-intensity exercise and its influence in combined physical and cognitive training.

## 7. Conclusions

Our study revealed that incorporating cognitive training into an exercise program may offer additional benefits to overall cognitive function in comparison with exercise-only programs, and similar additional benefits across exercise types. Given the complexity of multicomponent exercises, further studies should focus on combining cognitive training with a single exercise type, such as either aerobic or resistance. In the current SRMA, we observed no significant effect of combined exercises over exercise alone on BDNF. However, the evidence is insufficient to draw definitive conclusions regarding the effect of combined interventions on inflammatory and neuroprotective biomarkers. To develop targeted exercise programs that prevent cognitive decline in aging, it is essential to understand the biochemical mechanisms underlying the combined interventions that affect cognition. Therefore, further studies should include biochemical markers alongside cognitive assessments and study the associations between changes in biochemical markers and cognitive function. Given the publication bias observed in overall effects, the simultaneous delivery method and multicomponent exercise analysis, and the small number of studies that included resistance exercise analysis, may cause overestimation of the pooled effect. Due to the shifting effect size observed after conducting sensitivity analysis on the results of the combined resistance exercise studies, along with the limited number of studies included in this analysis, we cannot draw a conclusion regarding the superiority of any type of exercise when combined with cognitive training. Therefore, these results should be interpreted with caution.

## Figures and Tables

**Table 1:** Summary of the study characteristics.

Study Name	Study Population and Study Design	Age and Sample Size	Exercise Type with Cognitive Stimulation	Comparison Group	Exercise Intensity	Duration	Biomarkers	Cognitive Outcomes
Fabre et al., 2002 [52]	Healthy older persons (RCT)	60–76 years old Combined (*n* = 8 (F:7, M:1)) Exercise (*n* = 8 (F:6, M:2))	Aerobic exercise and cognitive stimulation (sequential)	Aerobic exercise	Heart Rate (HR) corresponded to Ventilatory Threshold (VT)	2 Months/2 Sessions for exercise and 1 session for cognitive training per week/60 min for exercise and 90 min for cognitive training group per session	NR	Memory quotient Paired-associate learning DSFT Logical memory immediate recall Orientation General information Mental control Visual reproduction
Barnes et al., 2013 [53]	Older persons with cognitive complaints (RCT)	>65 years old Combined (*n* = 32 (F:20, M:12)) Exercise (*n* = 31 (F:21, M:10))	Multicomponent group exercise + computer-based cognitive games (sequentially)	Multicomponent exercise + cognitive control (educational videos)	60–75% HR_max_	12 weeks/3 sessions per week/60 min each session	NR	Composite score RAVLT Verbal Fluency DSST TMT A and B EFT, visuospatial function
Ansai et al., 2017 [50]	Older persons (non-randomized)	≥60 years old Combined (*n* = 39) Exercise (*n* = 41) Gender information NR	Multicomponent exercise with cognitive training (simultaneously)	Multicomponent exercise	NR	12 weeks/3 sessions per week/50 min each session	NR	MMSE MOCA Clock Drawing Test
Anderson-Hanley et al., 2018 [54]	Older persons with MCI and sMCI (RCT)	>50 years old Combined (*n* = 7 (F:43%)) Exercise (*n* = 6 (F:57%))	Aerobic exercise with cognitive training (exercise-score) (simultaneously)	Aerobic exercise (exercise-tour)	Aerobic exercise (Average HR = 102.9) Combined exercise (Average HR = 108.2)	6 months/2–5 sessions per week/20–45 min each session	BDNF, CRP IGF-1, IL-6, and VEGF	Color Trails ½ Stroop A/C DSBT/DSFT Immediate Verbal Memory Delayed Verbal Memory
Combourieu Donnezan et al., 2018 [55]	Older persons with MCI (RCT)	≥65 years old Combined (*n* = 20) Exercise (*n* = 14) Gender Information NR	Aerobic exercise with computer-based cognitive training (simultaneously)	Aerobic exercise (cycling)	60% HR_max_	12 weeks/2 sessions per week/1 h each session	NR	Matrix Reasoning Test Stroop: Task Switching (number) DSFT DSBT
Damirchi et al., 2018 [56]	Older persons with MCI (RCT)	60–85 years old females Combined (*n* = 13) Exercise (*n* = 11)	Multicomponent exercise with cognitive training program “Modified My Better Mind” (sequentially)	Multicomponent exercise (walking, resistance, and cardio exercise)	55–75% HRR for aerobic exercise 13–15 RPE for resistance and range of motion exercise	8 weeks/3 sessions per week/30–60 min for cognitive training, and the exercise part consists of the same duration	BDNF IRISIN	Working Memory Processing Speed Reaction Time Error Number
Rezola-Pardo et al., 2019 [57]	Older persons with cognitive impairment (RCT)	≥70 years old Combined (*n* = 35) Exercise (*n* = 33) Gender information for the analyzed group NR	Multicomponent exercise (strength and balance) with cognitive training (simultaneously)	Multicomponent exercise (strength and balance)	40–70% of 1 RM for strength exercise; Moderate intensity for balance training, and 5–6 RPE from the Borg CR10 scale	3 months/2 sessions per week/1 h each session	NR	MoCA Symbol Search Coding Semantic fluency Verbal fluency RAVLT TMT-A
Takeuchi et al., 2020 [58]	Older persons with and/or without MCI (RCT)	65–75 years old Combined (*n* = 30, (F: 18, M:12)) Exercise (*n* = 33 (F:21, M:12)	Aerobic exercise with cognitive training (simultaneously)	Aerobic exercise	40–50% HRR	12 weeks/3 sessions per week/60 min each session	NR	0-Back Accuracy 2-Back Accuracy RAPM Digit Span Logical Memory D-CAT FAB Symbol Search Digit Symbol SACT
Horata et al., 2021 [59]	Healthy older persons (RCT)	60–75 years old Combined (*n* = 16 (F:8, M:8)) Exercise (*n* = 16 (F:9, M:7))	Multicomponent exercise with cognitive training (simultaneously)	Multicomponent exercise	NR	6 weeks/2 sessions per week/60 min each session	NR	Stroop Tests SMMSE
Oliveira et al., 2021 [60]	Non-Robust older persons (RCT)	≥60 years old Combined (*n* = 7 (F:6, M:1)) Active control (*n* = 7 (F:2, M:5))	Exergame (multicomponent exercises) (simultaneously)	Active Control (same exercises as exergame group without cognitive stimulation)	NR	16 sessions/2 sessions per week/30–45 min each session	NR	Immediate Course Performance Maze Time Delayed Maze Time
Castaño et al., 2022 [61]	Older Persons (RCT)	≥60 years old Combined (*n* = 13 (F: 9, M:4)) Resistance (*n* = 12 (F: 9, M:3))	Resistance exercise with cognitive training (simultaneously)	Resistance exercise	60–70% of 1 RM	16 weeks/2 sessions per week/60 min each session	BDNF	SVF PVF TMT-A TMT-B SPMT
Li et al., 2022 [62]	Older persons with MCI (RCT)	≥65 years old Combined (*n* = 23 (F:16, M:7)) Tai Ji Quan (*n* = 22 (F:8, M:14))	Multicomponent exercise with cognitive training (Cognitively enhanced Tai Ji Quan) (simultaneously)	Multicomponent exercise (Standard Tai Ji Quan)	NR	16 weeks/2 sessions per week/60 min each session	NR	MoCA TMT-B DSFT Verbal Fluency
Béraud-Peigné et al., 2023 [63]	Cognitively healthy older persons (RCT)	≥60 years old Combined (*n* = 19) Exercise (*n* = 15) Gender Information NR for groups	Multicomponent exercise (Exergame) (simultaneously)	Multicomponent exercise	47% HRR for exergame; 44% HRR for exercise program)	12 weeks/2 sessions per week/60 min each session	NR	Zoo Map Test Plan Execution Spatial Span Backward Spatial Span Stroop Test (B1, B2, B3) Stroop Test Interference Score Mental Rotation Test TMT-A TMT-B TMT B-A
Chen et al., 2023 [51]	Older persons (Non-randomized)	60–84 years old Combined (*n* = 28 (F:17, M = 11) Exercise (*n* = 22 (F:13, M:9))	Multicomponent exercise (endurance, resistance, and stretching exercise) with cognitive training (simultaneously)	Multicomponent exercise (endurance, resistance, and stretching exercise)	6–10 RPE	12 weeks/2 sessions per week/90 min each session	NR	MMSE Chang Gung University Orthographical Fluency Test Taiwanese Frontal Assessment Battery
Uysal et al., 2023 [64]	Older persons with MCI (RCT)	≥60 years old Combined *n* = 12 (F:2, M:10)) Exercise (*n* = 12 (F:2, M:10))	Multicomponent exercise (aerobic and lower limb strength exercise) with dual task training (simultaneously)	Multicomponent exercise (aerobic and lower limb strength exercise)	Aerobic exercise started with 40–60% of HR_max_ and progressed to 50–75 of HR_max_ Lower limb strength started with 40% of 1 RM and progressed to 65–70% of 1 RM	12 weeks/3 sessions per week/30 min for aerobic, 30 min for dual task sessions/NR for strength sessions	NR	MMSE
Ayed et al., 2024 [65]	Older persons with MCI (RCT)	60–75 years old Combined (*n* = 15 (F:10, M:5)) Exercise (*n* = 15 (F:10, M:5))	Aerobic Exercise with cognitive training (simultaneously)	Aerobic exercise	≈ 60% of HR_max_	8 weeks/2 sessions per week/30–45 Minutes each session	NR	MMSE RL/RI-16 Letter fluency Category fluency Clock Test Stroop Test DSFT DSBT Hanoi Moves Hanoi Time
Baek et al., 2024 [66]	Older Persons with cognitive impairment (RCT)	≥65 years old Combined (*n* = 22 (F:15, M:7)) Exercise (*n* = 22 (F:14, M:7)	Resistance exercise with cognitive training (simultaneously)	Resistance exercise	12 RM	6 weeks/3 sessions per week/40 min each session	NR	MMSE
Biehl-Printes et al., 2024 [67]	Older persons (RCT)	≥60 years old Combined (*n* = 24 (F:21, M:3)) Exercise (*n* = 23 (F:21, M:2))	Aerobic exercise with map reading (Orienteering) (simultaneously)	Aerobic exercise (Hiking)	12–13 RPE	24 weeks/2 sessions per week/60 min each session	NR	Concentrated Attention Processing Speed (Beta 1 Codes ID) Executive Functions (Beta 2 Matrix Reasoning) Pictorial Memory Test-2
Wang et al., 2024 [68]	Older persons (RCT)	≥60 years old females Combined (*n* = 17) Exercise (*n* = 17)	Resistance exercise with cognitive training (simultaneously)	Resistance exercise	10 RM	8 weeks/3 sessions per week/60 min each session	NR	TMT DST DSST
Ismail and Yusof, 2025 [69]	Older persons (RCT)	55–80 years old Combined (*n* = 14) Exercise (*n* = 14) Gender information NR	Multicomponent (Exergames) (simultaneously)	Multicomponent exercise without exergames	Moderate intensity (data NR)	8 weeks/3 sessions per week/30 min each session	BDNF	MoCA TMT-B DSST
Meekum et al., 2025 [70]	Older persons with MCI (RCT)	≥65 years old Combined (*n* = 19 (F:18, M:1)) Exercise (*n* = 18 (F:16, M:2))	Aerobic Exercise with cognitive training (simultaneously)	Aerobic exercise	50–70% of HR_max_	12 weeks/3 sessions per week/60 min	NR	MoCA DSFT DSBT Stroop Color and Word Test TMT-A TMT-B TMT B-A
Silva et al., 2025 [71]	Older persons with cognitive decline (RCT)	≥65 years old Combined strength training (*n* = 56 (F:27, M:29)) Strength training (*n* = 23 (F:17, M:6)) Combined aerobic training (*n* = 34 (F:25, M:9)) Aerobic training (*n* = 41 (F:38, M:3))	Strength exercise combined with cognitive training Aerobic exercise combined with cognitive training (sequentially)	Strength exercise Aerobic exercise	Strength exercise 4/5 repetitions in reserve (75–85% HR_max_) Aerobic exercise (75–85% HR_max_)	12 Weeks/3 sessions per week/60 min each session Cognitive training lasts 20 min after the exercise sessions	NR	MoCA
de Almeida et al., 2026 [72]	Older persons (RCT)	50–80 years old Combined (*n* = 27, F: 65.2%) Exercise (*n* = 30, F:88.9%)	Multicomponent exercise with cognitive training (Sequentially)	Multicomponent exercise	6–7 RPE (Borg CR-10)	12 weeks/2 sessions per week/90 min each session	Cholesterol Glucose	MoCA Clock Drawing Test
Han et al., 2026 [41]	Older persons with cognitive impairment (RCT)	≥65 years old Combined exercise with 30 min cognitive training (*n* = 21, (F:13, M:8)) Combined Exercise with 15 min cognitive training (*n* = 21, (F:13, M:8)) Exercise (*n* = 21, (F:17, M:4))	Aerobic exercise with cognitive training (simultaneously)	Aerobic exercise	11–12 RPE	3 months/3 sessions per week/30 min each session	NR	MoCA MMSE

BDNF, Brain Derived Neurotrophic Factor; CRP, C-Reactive Protein; D-CAT, Digit Cancelation Test; DSBT, Digit Span Backward Test; DSFT, Digit Span Forward Test; DSST, Digit Symbol Substitution Test; EFT, Eriksen Flanker Test; F, Female; FAB, Frontal Assessment Battery; HR, Heart Rate; HRmax, Maximal Heart Rate; HRR, Heart Rate Reserve; IGF-1, Insulin Like Growth Factor-1; IL-6, Interleukin-6; M, Male; MCI, Mild Cognitive Impairment; MMSE, Mini Mental State Examination; MoCA, Montreal Cognitive Assessment Test; NR, Not Reported; PVF, Phonological Verbal Fluency; RAVLT, Rey Auditory Verbal Learning Test; RAPM, Ravens Advanced Progressive Matrix; RCT, Randomized Controlled Trial; RL/RI-16, 16-item Free and Cued Recall; RPE, Rate of Perceived Exertion; SACT, S-A Creativity Test; SVF, Semantic Verbal Fluency; SPMT, Scenery Picture Memory Test; TMT, Trail Making Test; VEGF, Vascular Endothelial Growth Factor; VT, Ventilatory Threshold.

## Data Availability

The dataset is available from the corresponding author, Kaan Akalp, upon request via e-mail.

## Supplementary Materials

The following supporting information can be downloaded at: https://www.mdpi.com/article/10.3390/healthcare14162600/s1. Supplementary Figure S1. Funnel Plot of the comparison of overall effects of exercise and combined intervention on cognitive function. Supplementary Figure S2. Meta-Analysis of the comparison between the overall effects of exercise and a simultaneously combined intervention. Supplementary Figure S3. Meta-Analysis of the comparison between overall effects of exercise and sequentially combined intervention. Supplementary Figure S4. Funnel Plot of comparison of overall effects of exercise and combined intervention on BDNF. Supplementary Figure S5. Funnel Plot of comparison of effects of aerobic exercise and combined aerobic exercise with cognitive training. Supplementary Figure S6. Funnel Plot of the comparison of effects of multicomponent exercise and combined exercise with cognitive training. Supplementary Figure S7. Funnel Plot of comparison effects of resistance exercise and combined exercise with cognitive training. Supplementary Table S1. PRISMA 2020 Checklist. Supplementary Table S2. Search Strategy for each database. Supplementary Table S3a. Sensitivity Analysis Results—Non-randomized studies and one arm from a multi-arm study removed. Supplementary Table S3b. Sensitivity Analysis Result—With pre-post correlation 0.3. Supplementary Table S3c. Sensitivity Analysis Result-With pre-post correlation 0.7. Supplementary Table S4. Cognitive tests and corresponding cognitive domains included in the systematic review and meta-analysis. Supplementary Table S5. Methodological quality scores of the included studies. References [108,109,110,111,112,113,114,115,116,117,118,119,120,121,122,123,124,125,126,127,128,129,130,131,132,133] are cited in the Supplementary Materials.

## Abbreviations

The following abbreviations are used in this manuscript:

1-RM	1 Repetition Maximum
BDNF	Brain-derived neurotrophic factor
CRP	C-Reactive Protein
D-CAT	Digit Cancellation Test
DSBT	Digit Span Backward Test
DSFT	Digit Span Forward Test
DSST	Digit Symbol Substitution Test
EFT	Eriksen Flanker Test
FAB	Frontal Assessment Battery
HR_max_	Maximal Heart Rate
HRR	Heart Rate Reserve
IGF-1	Insulin-like Growth Factor-1
IL-6	Interleukin-6
MCI	Mild Cognitive Impairment
MMSE	Mini-Mental State Examination
MoCA	Montreal Cognitive Assessment Test
NR	Not Reported
PVF	Phonological Verbal Fluency
RAVLT	Rey Auditory Verbal Learning Test
RAPM	Ravens Advanced Progressive Matrix
RL/RI-16	16-item Free and Cued Recall
RPE	Rate of Perceived Exertion
SVF	Semantic Verbal Fluency
SPMT	Scenery Picture Memory Test
TMT	Trail Making Test
VEGF	Vascular Endothelial Growth Factor
VT	Ventilatory Threshold
