# Supplementary material for: Effects of Combined Exercise Interventions on Cognition in Older Persons: A Systematic Review and Meta-Analysis
Source: Healthcare (Basel). 2026 Aug 18;14(16):2600. doi: 10.3390/healthcare14162600 (PMC13511800; doi:10.3390/healthcare14162600)
Supplement: Supplementary file 1 [file healthcare-14-02600-s001.zip › Supplementary Table S5 .pdf]

Supplementary Table S5. Methodological quality scores of the included studies.

Study Names	T.S.	Q1	Q2	Q3	Q4	Q5	Q6	Q7	Q8	Q9	Q10	Q11	Q12	Q13	Q14	Q15	Q16	Q17	Q18	Q19	Q20	Q21	Q22	Q23	Q24	Q25	Q26	Q27
**Fabre et al. (2002)**	17	1	1	1	1	1	1	1	0	0	1	0	1	0	0	1	1	1	1	0	1	0	0	1	0	1	1	0
**Barnes et al. (2013)**	22	1	1	1	1	1	1	1	1	0	1	0	1	1	1	0	1	1	1	1	1	0	0	1	1	1	1	1
**Ansai et al. (2017)**	19	1	1	1	1	2	1	1	0	0	1	1	1	0	0	0	1	1	1	1	1	1	0	0	0	1	0	1
**Anderson-Hanley et al. (2018)**	21	1	1	1	1	2	1	1	1	1	1	1	0	0	0	0	1	1	1	0	1	1	0	1	0	1	1	1
**Combourieu Donnezan et al. (2018)**	21	1	1	1	1	1	1	1	1	1	1	1	1	0	0	0	1	1	1	0	1	1	0	1	0	1	1	1
**Damirchi et al. (2018)**	22	1	1	1	1	2	1	1	0	1	1	1	1	0	0	0	1	1	1	1	1	1	0	1	0	1	1	1
**Rezola-Pardo et al. (2019)**	25	1	1	1	1	1	1	1	1	1	1	1	1	1	1	1	1	1	1	0	1	1	0	1	1	1	1	1
**Takeuchi et al. (2020)**	20	1	1	1	1	2	1	0	1	1	0	0	1	0	1	1	1	1	1	1	1	1	0	1	0	1	0	0

**Horata et al. (2021)**	22	1	1	1	1	2	1	1	1	0	1	1	1	1	0	0	1	1	1	1	1	1	0	1	0	1	0	1
**Oliveira et al. (2021)**	17	1	1	1	1	1	1	1	0	0	1	1	1	0	1	0	1	1	1	0	1	0	0	1	1	0	0	0
**Castano et al. (2022)**	18	1	1	1	1	1	1	1	0	1	1	0	0	1	0	1	1	1	1	1	0	0	0	1	0	0	1	1
**Li et al. (2022)**	26	1	1	1	1	2	1	1	1	1	1	1	0	1	0	1	1	1	1	1	1	1	1	1	1	1	1	1
**Uysal et al. (2023)**	22	1	1	1	1	1	1	1	1	1	1	0	1	0	1	1	1	1	1	0	1	0	0	1	1	1	1	1
**Beraud-Peigne et al. (2023)**	22	1	1	1	1	2	1	1	0	1	1	1	1	0	1	0	1	0	1	1	1	1	0	1	1	1	0	1
**Chen et al. (2023)**	20	1	1	1	1	2	1	1	0	0	1	1	1	1	0	0	1	1	1	1	1	1	0	0	0	1	0	1
**Baek et al. (2024)**	24	1	1	1	1	2	1	1	0	1	1	1	0	1	0	1	1	1	1	1	1	1	1	0	1	1	1	1
**Biehl-Printes et al. (2024)**	22	1	1	1	1	2	1	1	1	0	1	1	1	1	0	0	1	1	1	1	1	0	0	1	0	1	1	1
**Ben Ayed et al. (2024)**	17	1	1	1	1	2	1	1	0	0	0	1	1	0	0	0	1	1	1	0	1	1	0	1	0	0	0	1
**Wang et al. (2024)**	21	1	1	1	1	2	1	1	0	1	1	0	0	0	0	0	1	1	1	1	1	1	1	1	0	1	1	1
**Ismail et al. (2025)**	20	1	1	0	1	1	1	1	0	1	1	1	0	1	0	0	1	1	1	1	1	1	1	1	0	0	1	1

**Meekum et al. (2025)**	20	1	1	1	1	2	1	1	0	1	1	0	1	0	1	0	1	1	1	0	1	0	0	1	1	1	1	0
**Silva et al. (2025)**	20	1	1	1	1	1	1	1	1	1	1	1	0	0	0	0	1	1	1	0	1	0	0	1	1	1	1	1
**De Almeida et al. (2026)**	23	1	1	1	1	2	1	1	0	1	1	1	1	1	0	0	1	1	1	1	1	1	0	1	1	1	1	0
**Han et al. (2026)**	23	1	1	1	1	2	1	1	0	1	1	0	0	0	0	1	1	1	1	1	1	1	1	1	1	1	1	1

T.S. = Total Score; Q = Question; 1 = yes (due to three options for question number five, 1 indicates “partial” option for question number 5); 0 = no or unable to determine; 2 = yes.

References

Anderson-Hanley, C., Barcelos, N.M., Zimmerman, E.A., Gillen, R.W., Dunnam, M., Cohen, B.D., et al. (2018). The Aerobic and Cognitive Exercise Study (ACES) for Community-Dwelling Older Adults With or At-Risk for Mild Cognitive Impairment (MCI): Neuropsychological, Neurobiological and Neuroimaging Outcomes of a Randomized Clinical Trial. *Frontiers in Aging Neuroscience* 10. doi: 10.3389/fnagi.2018.00076.

Ansai, J.H., de Andrade, L.P., Buto, M.S.D., Barroso, V.D., Farche, A.C.S., Rossi, P.G., et al. (2017). Effects of the Addition of a Dual Task to a Supervised Physical Exercise Program on Older Adults' Cognitive Performance. *Journal of Aging and Physical Activity* 25(2), 234-239. doi: 10.1123/japa.2016-0094.

Ayed, I.B., Aouichaoui, C., Ammar, A., Naija, S., Tabka, O., Jahrami, H., et al. (2024). Mid-Term and Long-Lasting Psycho-Cognitive Benefits of Bidomain Training Intervention in Elderly Individuals with Mild Cognitive Impairment. *European Journal of Investigation in Health Psychology and Education* 14(2), 284-298. doi: 10.3390/ejihpe14020019.

Baek, J.E., Hyeon, S.J., Kim, M., Cho, H.Y., and Hahm, S.C. (2024). Effects of dual-task resistance exercise on cognition, mood, depression, functional fitness, and activities of daily living in older adults with cognitive impairment: a single-blinded, randomized controlled trial. *Bmc Geriatrics* 24(1). doi: 10.1186/s12877-024-04942-1.

Barnes, D.E., Santos-Modesitt, W., Poelke, G., Kramer, A.F., Castro, C., Middleton, L.E., et al. (2013). The mental activity and exercise (MAX) trial: A randomized controlled trial to enhance cognitive function in older adults. *JAMA Internal Medicine* 173(9), 797-804. doi: 10.1001/jamainternmed.2013.189.

Béraud-Peigné, N., Maillot, P., and Perrot, A. (2023). The effects of a new immersive multidomain training on cognitive, dual-task and physical functions in older adults. *GeroScience*. doi: 10.1007/s11357-023-00952-w.

Biehl-Printes, C., Irigaray, T.Q., Dornelles, J.O.F., and Baptista, R.R. (2024). Unraveling the unparalleled benefits of orienteering versus hiking on gait performance and cognition: A randomized clinical trial. *Archives of Gerontology and Geriatrics* 117. doi: 10.1016/j.archger.2023.105201.

Castaño, L.A.A., Castillo de Lima, V., Barbieri, J.F., de Lucena, E.G.P., Gáspari, A.F., Arai, H., et al. (2022). Resistance Training Combined With Cognitive Training Increases Brain Derived Neurotrophic Factor and Improves Cognitive Function in Healthy Older Adults. *Front Psychol* 13, 870561. doi: 10.3389/fpsyg.2022.870561.

Chen, Y.L., Tseng, C.H., Lin, H.T., Wu, P.Y., and Chao, H.C. (2023). Dual-task multicomponent exercise-cognitive intervention improved cognitive function and functional fitness in older adults. *Aging Clinical and Experimental Research* 35(9), 1855-1863. doi: 10.1007/s40520-023-02481-0.

Combourieu Donnezan, L., Perrot, A., Belleville, S., Bloch, F., and Kemoun, G. (2018). Effects of simultaneous aerobic and cognitive training on executive functions, cardiovascular fitness and functional abilities in older adults with mild cognitive impairment. *Mental Health and Physical Activity* 15, 78-87. doi: 10.1016/j.mhpa.2018.06.001.

Damirchi, A., Hosseini, F., and Babaei, P. (2018). Mental Training Enhances Cognitive Function and BDNF More Than Either Physical or Combined Training in Elderly Women With MCI: A Small-Scale Study. *American Journal of Alzheimers Disease and Other Dementias* 33(1), 20-29. doi: 10.1177/1533317517727068.

de Almeida, M.L., Rodrigues, G.D., Sobrinho, A.C.D., Prado, L.D., Rodrigues, K.P., and Bueno, C.R. (2026). Cognitive Training Combined with Multicomponent Exercise Improves Biopsychosocial Outcomes in Healthy Older Adults: a Randomized Trial. *Journal of Cognitive Enhancement* 10(1), 65-80. doi: 10.1007/s41465-026-00347-7.

Fabre, C., Chamari, K., Mucci, P., Massé-Biron, J., and Préfaut, C. (2002). Improvement of cognitive function by mental and/or individualized aerobic training in healthy elderly subjects. *Int J Sports Med* 23(6), 415-421. doi: 10.1055/s-2002-33735.

Han, C.L., Lv, J.J., Sun, R.F., Zhen, Q.X., Song, L., Wu, J.L., et al. (2026). Aerobic exercise combined with varied duration cognitive training to improve age-related cognitive impairment: A randomized controlled trial. *Geriatric Nursing* 70. doi: 10.1016/j.gerinurse.2026.103981.

Horata, E.T., Cetin, S.Y., and Erel, S. (2021). Effects of individual progressive single- and dual-task training on gait and cognition among older healthy adults: a randomized-controlled comparison study. *European Geriatric Medicine* 12(2), 363-370. doi: 10.1007/s41999-020-00429-5.

Ismail, N.A., and Yusof, H.A. (2025). The effect of brain-derived neurotrophic factor (BDNF) Val66Met gene polymorphism on cognitive performance of aging brain in response to an 8-week of exergames training. *Sport Sciences for Health* 21(1), 363-378. doi: 10.1007/s11332-024-01267-9.

Li, F., Harmer, P., Fitzgerald, K., and Winters-Stone, K. (2022). A cognitively enhanced online Tai Ji Quan training intervention for community-dwelling older adults with mild cognitive impairment: A feasibility trial. *BMC Geriatrics* 22(1). doi: 10.1186/s12877-021-02747-0.

Meekum, S., Yuenyongchaiwat, K., Luangpon, N., Keawutan, P., and Kooncumchoo, P. (2025). Enhancing Global Cognition and Executive Functions Through Water-Based Exercise in Mild Cognitive Impairment: A Randomized Controlled Trial. *Life-Basel* 15(3), 13. doi: 10.3390/life15030420.

Oliveira, L.M., Souza, E., Alves, M.R., Carneiro, L.S.F., Fagundes, D.F., de Paula, A.M.B., et al. (2021). 2D Virtual Reality-Based Exercise Improves Spatial Navigation in Institutionalized Non-robust Older Persons: A Preliminary Data Report of a Single-Blind, Randomized, and Controlled Study. *Frontiers in Neurology* 11. doi: 10.3389/fneur.2020.609988.

Rezola-Pardo, C., Arrieta, H., Gil, S.M., Zarrazquin, I., Yanguas, J.J., López, M.A., et al. (2019). Comparison between multicomponent and simultaneous dual-task exercise interventions in long-term nursing home residents: the Ageing-ONDUAL-TASK randomized controlled study. *Age and Ageing* 48(6), 817-823. doi: 10.1093/ageing/afz105.

Silva, A.F., Clemente, F.M., Roriz, M.S., Azevedo, J.A., Jovanovic, O., Adamovic, M., et al. (2025). The Effect of Aerobic or Strength Training in Elderly with Cognitive Decline: The Fit4Alz Project. *Journal of Sports Science and Medicine* 24(1), 172-186. doi: 10.52082/jssm.2025.172.

Takeuchi, H., Magistro, D., Kotozaki, Y., Motoki, K., Nejad, K.K., Nouchi, R., et al. (2020). Effects of Simultaneously Performed Dual-Task Training with Aerobic Exercise and Working Memory Training on Cognitive Functions and Neural Systems in the Elderly. *Neural Plasticity* 2020. doi: 10.1155/2020/3859824.

Uysal, I., Basar, S., Aysel, S., Kalafat, D., and Büyüksünnetçi, A.O. (2023). Aerobic exercise and dual-task training combination is the best combination for improving cognitive status, mobility and physical performance in older adults with mild cognitive impairment. *Aging Clinical and Experimental Research* 35(2), 271-281. doi: 10.1007/s40520-022-02321-7.

Wang, Y.X., Zhang, C., Wang, B., Zhang, D.X., and Song, X.B. (2024). Comparative effects of cognitive and instability resistance training versus instability resistance training on balance and cognition in elderly women. *Scientific Reports* 14(1). doi: 10.1038/s41598-024-77536-x.
