# Supplementary material for: Effects of Combined Exercise Interventions on Cognition in Older Persons: A Systematic Review and Meta-Analysis
Source: Healthcare (Basel). 2026 Aug 18;14(16):2600. doi: 10.3390/healthcare14162600 (PMC13511800; doi:10.3390/healthcare14162600)
Supplement: Supplementary file 1 [file healthcare-14-02600-s001.zip › Supplementary Figures .pdf]

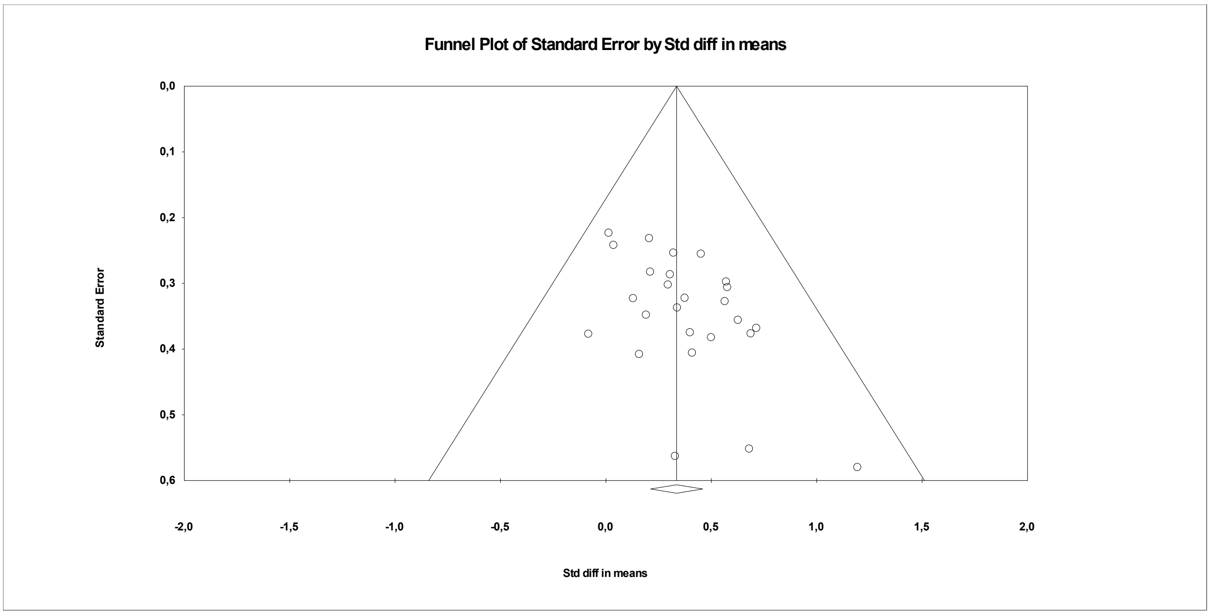

Supplementary Figure S1. Funnel Plot of the comparison of overall effects of exercise and combined intervention on cognitive function.

a

Simultaneous Method

Study name	Outcome	Std diff in means	Standard error	Variance	Lower limit	Upper limit	Z-Value	p-Value
		Statistics for each study						
Combourieu et all (2018)	Combined	0,133	0,324	0,105	-0,502	0,768	0,411	0,681
Rezola-Padro et al (2019)	Combined	0,040	0,243	0,059	-0,436	0,516	0,165	0,869
Oliveira et al(2021)	Combined	1,197	0,581	0,337	0,058	2,335	2,060	0,039
Li et al(2022)	Combined	0,580	0,307	0,094	-0,022	1,182	1,888	0,059
Beraud-Peigne et al(2023)	Combined	0,195	0,349	0,122	-0,490	0,879	0,557	0,577
Uysal et al(2023)	MMSE	0,162	0,409	0,167	-0,640	0,963	0,395	0,693
Biehl-Printes et al(2024)	Combined	0,574	0,299	0,089	-0,011	1,160	1,924	0,054
Ben Ayed et al(2024)	Combined	0,691	0,378	0,143	-0,049	1,431	1,830	0,067
Meekum et al(2025)	Combined	0,342	0,338	0,114	-0,321	1,005	1,011	0,312
Anderson et al (2018)	Combined	0,332	0,564	0,318	-0,773	1,437	0,589	0,556
Castano et al. (2022)	Combined	0,413	0,407	0,166	-0,385	1,210	1,014	0,310
Ansai et al(2017)	Combined	0,017	0,224	0,050	-0,423	0,457	0,077	0,939
Baek et al(2024)	MMSE	0,298	0,303	0,092	-0,296	0,893	0,984	0,325
Chen et al(2023)	Combined	0,308	0,288	0,083	-0,256	0,872	1,071	0,284
Han et al(2026)a	Combined	0,568	0,328	0,108	-0,076	1,212	1,729	0,084
Han et al(2026)b	Combined	0,378	0,323	0,105	-0,256	1,012	1,169	0,243
Horata et al(2021)	Combined	0,718	0,369	0,136	-0,006	1,441	1,944	0,052
Ismail et al(2025)	Combined	-0,079	0,378	0,143	-0,820	0,662	-0,209	0,834
Takeuchi et al(2020)	Combined	0,324	0,255	0,065	-0,175	0,823	1,272	0,203
Wang et al(2024)	Combined	0,630	0,357	0,127	-0,069	1,330	1,765	0,078
Pooled		0,331	0,073	0,005	0,189	0,474	4,565	0,000
Prediction Interval								

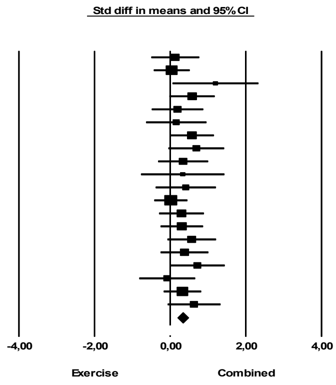

b

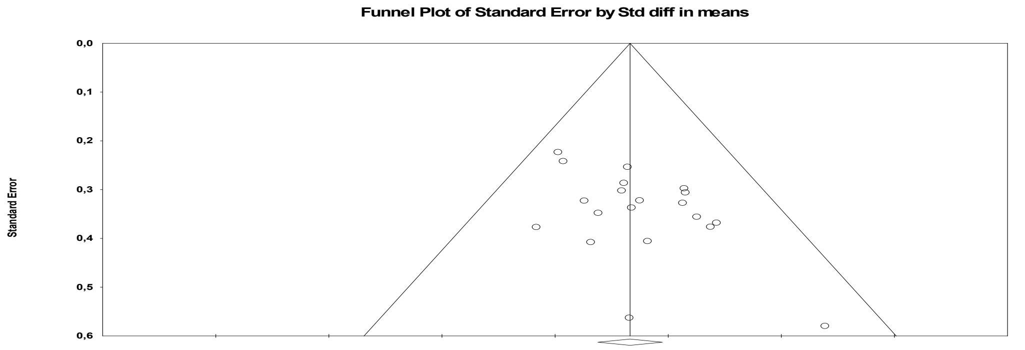

Supplementary Figure S2. Meta-Analysis of the comparison between the overall effects of exercise and a simultaneously combined intervention. **a.** Forest Plot of the comparison of overall effects of exercise and simultaneously combined intervention on cognitive function; **b.** Funnel Plot of the comparison of overall effects of exercise and a simultaneously combined intervention on cognitive function. (Silva et al., 2025a and Silva et al., 2025b indicate the same study with two different types of exercise; Han et al., 2026a and Han et al., 2026b indicate the same study with two different combined interventions).

a

Sequential Method

Study name	Outcome	Std diff in means	Standard error	Variance	Lower limit	Upper limit	Z-Value	p-Value
Febre et al(2002)	Combined	0,684	0,553	0,306	-0,400	1,767	1,237	0,216
Damirchi et al(2018)	Combined	0,403	0,376	0,141	-0,334	1,139	1,071	0,284
Silva et al(2025)a	MoCA	0,455	0,256	0,066	-0,048	0,957	1,774	0,076
Silva et al(2025)b	MoCA	0,209	0,233	0,054	-0,247	0,665	0,899	0,369
Barnes et al(2013)	Combined	0,214	0,284	0,080	-0,342	0,770	0,755	0,450
de Almeida et al(2026)	Combined	0,503	0,383	0,147	-0,248	1,254	1,312	0,189
Pooled		0,347	0,126	0,016	0,101	0,593	2,760	0,006
Prediction Interval								

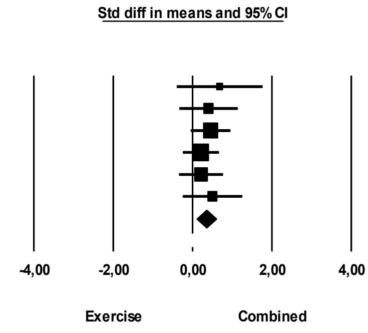

b

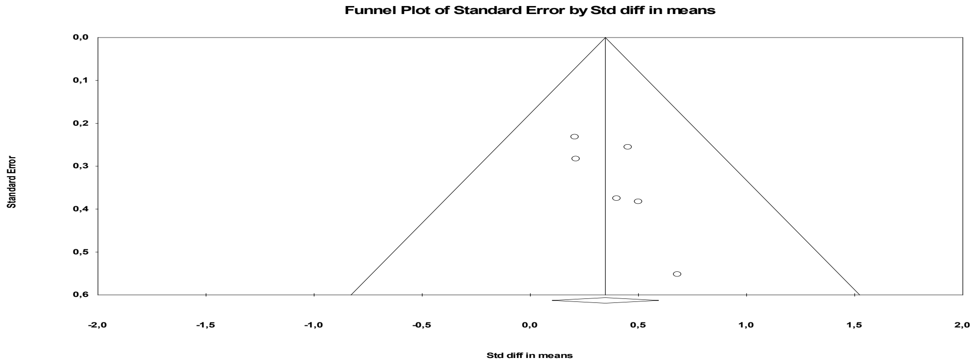

Supplementary Figure S3. Meta-Analysis of the comparison between overall effects of exercise and sequentially combined intervention. **a.** Forest Plot of the comparison of overall effects of exercise and sequentially combined intervention on cognitive function; **b.** Funnel Plot of the comparison of overall effects of exercise and sequentially combined intervention on cognitive function. (Silva et al., 2025a and Silva et al., 2025b indicate the same study with two different types of exercise).

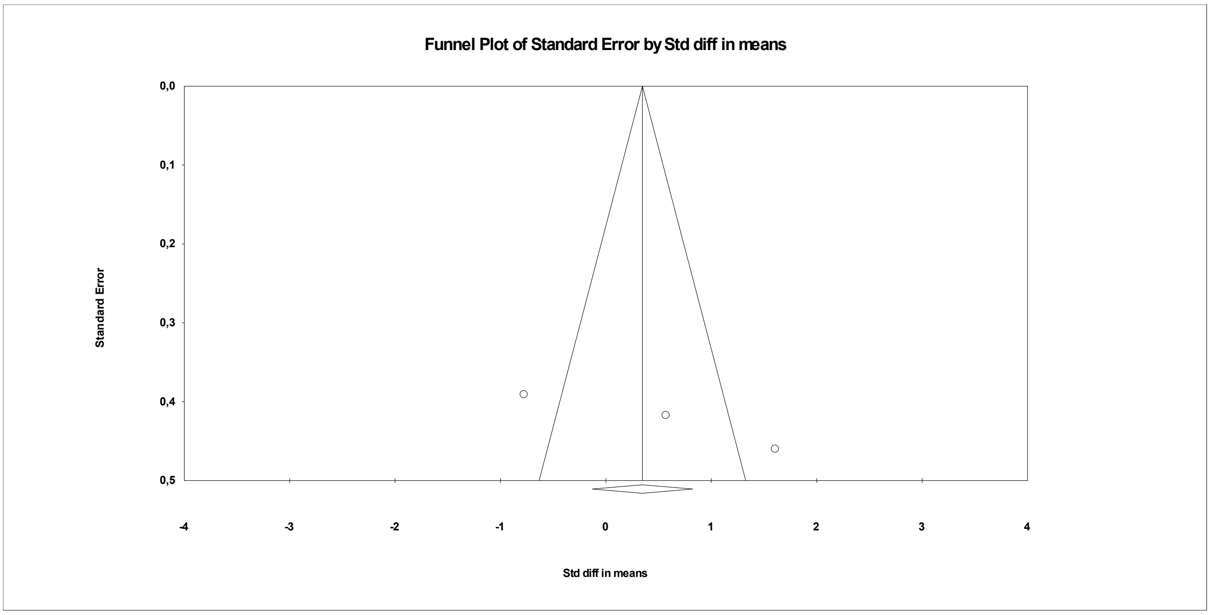

Supplementary Figure S4. Funnel Plot of comparison of overall effects of exercise and combined intervention on BDNF

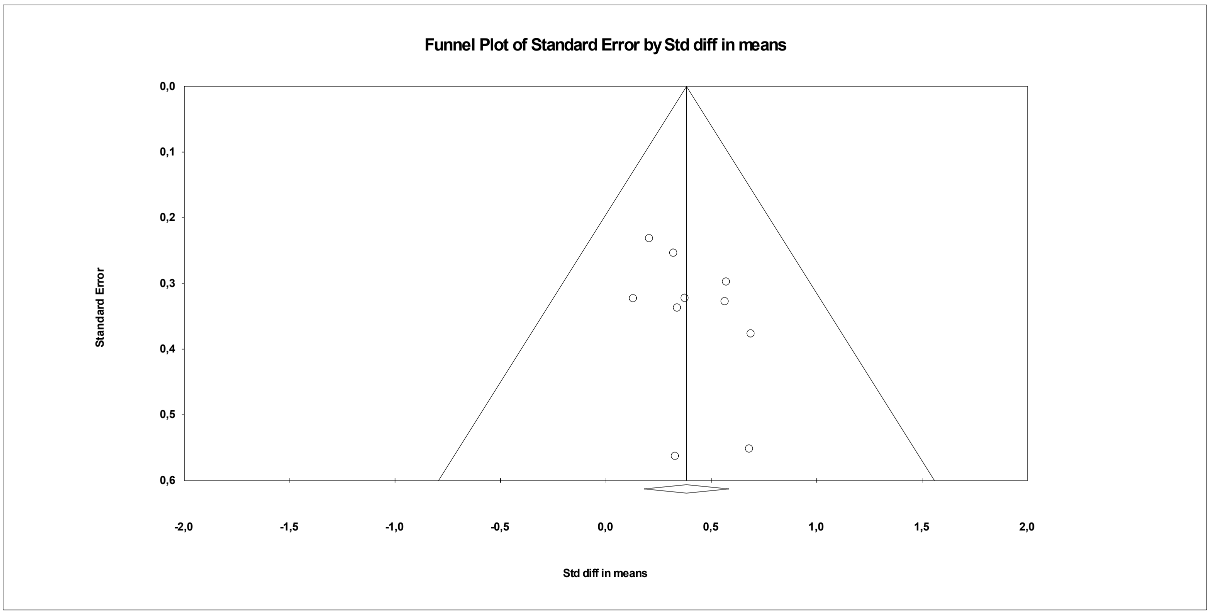

Supplementary Figure S5. Funnel Plot of comparison of effects of aerobic exercise and combined aerobic exercise with cognitive training.

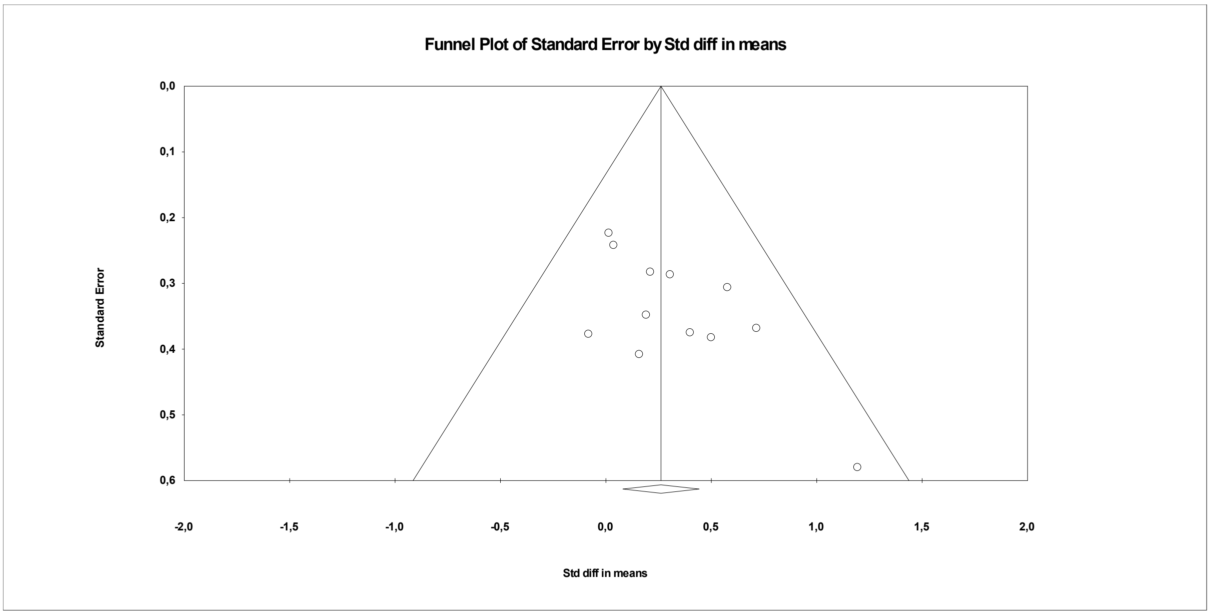

Supplementary Figure S6. Funnel Plot of the comparison of effects of multicomponent exercise and combined exercise with cognitive training.

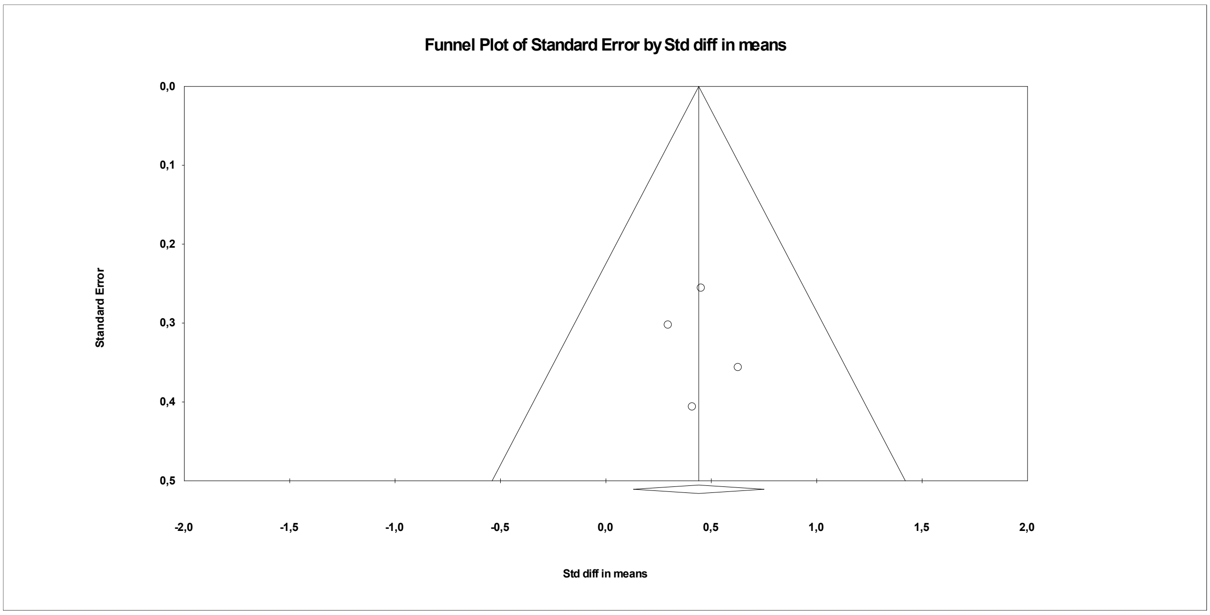

Supplementary Figure S7. Funnel Plot of comparison effects of resistance exercise and combined exercise with cognitive training.
