# Supplementary material for: Effects of Combined Exercise Interventions on Cognition in Older Persons: A Systematic Review and Meta-Analysis
Source: Healthcare (Basel). 2026 Aug 18;14(16):2600. doi: 10.3390/healthcare14162600 (PMC13511800; doi:10.3390/healthcare14162600)
Supplement: Supplementary file 1 [file healthcare-14-02600-s001.zip › Supplementary Table S2.pdf]

Supplementary Table S2. Search Strategy for each database

Database	**Search String**	**Filters/Limits**	**Records identified**
PubMed (All Fields)	(((("Older Adults" OR "Older Persons" OR "Elderly" OR "Aging" OR "MCI" OR "Mild Cognitive Impairment")) AND (("Dual Task" OR "Multimodal" OR "Cognitive Training" OR "Cognitive Stimulation" OR "Exergame" OR "Exergaming"))) AND (("Physical Exercise" OR "Aerobic Exercise" OR "Resistance Exercise" OR "Exercise" OR "Physical Activity"))) AND (("Mental health" OR "Cognitive assessment" OR "Memory" OR "Neurogenesis" OR "Neuroplasticity" OR "Brain health" OR "BDNF" OR "IGF-1" OR "IL-6" OR "TNF-alpha" OR "VEGF" OR "Irisin" OR "NFL" OR "Cognition" OR "MMSE" OR "MoCA" OR "Cognitive Function"))	clinical study, clinical trial, randomized controlled trial, English, and Humans	430
Web of Science (All Fields)	("Older Adults" OR "Older Persons" OR "Elderly" OR "Aging" OR "MCI" OR "Mild Cognitive Impairment") AND ("Dual Task" OR "Multimodal" OR "Cognitive Training" OR "Cognitive Stimulation" OR "Exergame" OR "Exergaming") AND (" Physical Exercise " OR "Aerobic Exercise " OR "Resistance Exercise " OR " Exercise " OR " Physical Activity") AND ("Mental health" OR "Cognitive assessment" OR "Memory" OR "Neurogenesis" OR "Neuroplasticity" OR "Brain health" OR "BDNF" OR "IGF-1" OR "IL-6" OR "TNF-alpha" OR "VEGF" OR "Irisin" OR "NFL" OR "Cognition" OR "MMSE" OR "MoCA" OR "Cognitive Function")	article and English	1354
Scopus	TITLE-ABS-KEY (("Older Adults" OR "Older Persons" OR "Elderly" OR "Aging" OR "MCI" OR "Mild Cognitive Impairment") AND ("Dual Task" OR "Multimodal" OR "Cognitive Training" OR "Cognitive Stimulation" OR "Exergame" OR "Exergaming") AND (" Physical Exercise " OR " Aerobic Exercise " OR " Resistance Exercise " OR " Exercise " OR " Physical Activity") AND ("Mental health" OR "Cognitive assessment" OR "Memory" OR "Neurogenesis" OR "Neuroplasticity" OR "Brain health" OR "BDNF" OR "IGF-1" OR "IL-6" OR "TNF-alpha" OR "VEGF" OR "Irisin" OR "NFL" OR "Cognition" OR "MMSE" OR "MoCA" OR "Cognitive Function"))	human, article, and English	1598
PsycInfo (All Fields)	("Older Adults" OR "Older Persons" OR "Elderly" OR "Aging" OR "MCI" OR "Mild Cognitive Impairment") AND ("Dual Task" OR "Multimodal" OR "Cognitive Training" OR "Cognitive Stimulation" OR "Exergame" OR "Exergaming") AND (" Physical Exercise " OR "Aerobic Exercise " OR "Resistance Exercise " OR " Exercise " OR " Physical Activity") AND ("Mental health" OR "Cognitive assessment" OR "Memory" OR "Neurogenesis" OR "Neuroplasticity" OR "Brain health" OR "BDNF" OR "IGF-1" OR "IL-6" OR "TNF-alpha" OR "VEGF" OR "Irisin" OR "NFL" OR "Cognition" OR "MMSE" OR "MoCA" OR "Cognitive Function")	article, exercise, aging, humans	419
