# Supplementary material for: Effects of Combined Exercise Interventions on Cognition in Older Persons: A Systematic Review and Meta-Analysis
Source: Healthcare (Basel). 2026 Aug 18;14(16):2600. doi: 10.3390/healthcare14162600 (PMC13511800; doi:10.3390/healthcare14162600)
Supplement: Supplementary file 1 [file healthcare-14-02600-s001.zip › Supplementary Table S3 Sensitivity Analyses.pdf]

Supplementary Table S3a. Sensitivity Analysis Results—Non-randomized studies and one arm from a multi-arm study removed (Ansai et al., 2017; Chen et al., 2023; Han et al., 2026)

**Global Effects of Exercise and Combined Intervention on Cognitive Function**							
ES	p-value	95% CI	Z-value	Q-value	I^2	τ^2	τ
0.365	<0.001	0.230 to 0.500	5.305	11.213	-	-	-
**Effects of Aerobic Exercise with Combined Intervention on Cognitive Function**							
ES	p-value	95% CI	Z-value	Q-value	I^2	τ^2	τ
0.383	<0.001	0.172 to 0.594	3.552	2.920	-	-	-
**Effects of Multicomponent Exercise with Combined Intervention on Cognitive Function**							
ES	p-value	95% CI	Z-value	Q-value	I^2	τ^2	τ
0.311	0.004	0.099 to 0.524	2.877	7.285	-	-	-
**Effects of Resistance Exercise with Combined Intervention on Cognitive Function**							
ES	p-value	95% CI	Z-value	Q-value	I^2	τ^2	τ
0.440	0.005	0.130 to 0.750	2.784	0.510	-	-	-

Supplementary Table 3b. Sensitivity Analysis Result—With pre-post correlation 0.3

**Global Effects of Exercise and Combined Intervention on Cognitive Function**							
**ES**	p-value	95% CI	Z-value	Q-value	I^2	τ^2	τ
**0.290**	<0.001	0.167 to 0.412	4.362	10.435	-	-	-
**Effects of Aerobic Exercise with Combined Intervention on Cognitive Function**							
**ES**	p-value	95% CI	Z-value	Q-value	I^2	τ^2	τ
**0.328**	0.001	0.129 to 0.528	3.223	2.324	-	-	-
**Effects of Multicomponent Exercise with Combined Intervention on Cognitive Function**							
ES	p-value	95% CI	Z-value	Q-value	I^2	τ^2	τ
0.229	0.012	0.049 to 0.409	2.499	6.846	-	-	-
**Effects of Resistance Exercise with Combined Intervention on Cognitive Function**							
ES	p-value	95% CI	Z-value	Q-value	I^2	τ^2	τ
0.376	0.017	0.068 to 0.685	2.389	0.381	-	-	-

Supplementary Table 3c. Sensitivity Analysis Result-With pre-post correlation 0.7

**Global Effects of Exercise and Combined Intervention on Cognitive Function**							
ES	p-value	95% CI	Z-value	Q-value	I^2	τ^2	τ
0.415	<0.001	0.291 to 0.540	6.537	19.523	-	-	-

**Effects of Aerobic Exercise with Combined Intervention on Cognitive Function**							
ES	p-value	95% CI	Z-value	Q-value	I^2	τ^2	τ
0.479	<0.001	0.277 to 0.681	4.644	4.240	-	-	-
**Effects of Multicomponent Exercise with Combined Intervention on Cognitive Function**							
ES	p-value	95% CI	Z-value	Q-value	I^2	τ^2	τ
0.325	0.001	0.130 to 0.521 (PI -0.002 to 0.653)	3.265	12.197	9.817	0.012	0.108
**Effects of Resistance Exercise with Combined Intervention on Cognitive Function**							
ES	p-value	95% CI	Z-value	Q-value	I^2	τ^2	τ
0.556	<0.001	0.243 to 0.869	3.485	0.772	-	-	-

ES, Effect Size (Standard Difference in Mean); CI, Confidence Interval; PI, Prediction Interval.

Ansai, J.H., de Andrade, L.P., Buto, M.S.D., Barroso, V.D., Farche, A.C.S., Rossi, P.G., et al. (2017). Effects of the Addition of a Dual Task to a Supervised Physical Exercise Program on Older Adults' Cognitive Performance. *Journal of Aging and Physical Activity* 25(2), 234-239. doi: 10.1123/japa.2016-0094.

Chen, Y.L., Tseng, C.H., Lin, H.T., Wu, P.Y., and Chao, H.C. (2023). Dual-task multicomponent exercise-cognitive intervention improved cognitive function and functional fitness in older adults. *Aging Clinical and Experimental Research* 35(9), 1855-1863. doi: 10.1007/s40520-023-02481-0.

Han, C.L., Lv, J.J., Sun, R.F., Zhen, Q.X., Song, L., Wu, J.L., et al. (2026). Aerobic exercise combined with varied duration cognitive training to improve age-related cognitive impairment: A randomized controlled trial. *Geriatric Nursing* 70. doi: 10.1016/j.gerinurse.2026.103981.
