# Supplementary material for: Effects of Combined Exercise Interventions on Cognition in Older Persons: A Systematic Review and Meta-Analysis
Source: Healthcare (Basel). 2026 Aug 18;14(16):2600. doi: 10.3390/healthcare14162600 (PMC13511800; doi:10.3390/healthcare14162600)
Supplement: Supplementary file 1 [file healthcare-14-02600-s001.zip › Supplementary Table S4.pdf]

Supplementary Table 4. Cognitive tests and corresponding cognitive domains included in the systematic review and meta-analysis

Global Cognitive Function	Attention	Executive Function	Processing Speed	Memory	Other
Mini Mental State Examination (+)[1-8]	Digit Cancellation task (+)[9, 10]	Trail Making Test A and B (-)[11-17]	Wechsler Adult Intelligence Scale Digit Symbol Substitution Test [11, 16, 18] (+)	Wechsler Adult Intelligence Memory Scale Memory Quotient (+)[18, 19]	Useful Field of View (Visuospatial Function) (-)[11, 20]
Montreal Cognitive Assessment (+)[2, 8, 13, 16, 17, 21-24]	Wechsler Adult Intelligence Scale Digit Span Forward(+)[22]	Eriksen Flanker Test Congruent and Incongruent Reaction Time (-)[11, 25]	Wechsler Adult Intelligence Scale Digit-Symbol Coding subtest (+)[13, 18, 26]	Rey Auditory Verbal Learning Test (+)[11, 13, 27]	Clock Drawing Test (-)[2, 6, 24, 28]
	Concentrated Attention Test (+)[29, 30]	Color Trials (+)[31]	BETA III General Non-Verbal Intelligence Test- BETA 1 Codes ID (+)[29, 32]	Immediate Verbal Memory (+)[31, 33]	Semantic Fluency (+)[13, 34]
	Trail Making Test (-)[35]	Stroop Color, word and A/C Word Test, Interference (-)[3, 6, 15, 17, 26, 31, 35-37]		Delayed Verbal Memory(+)[31, 33]	Verbal Fluency (+)[13, 14, 34]
		Digit Span Forward and Backward (+)[6, 10, 15, 17, 18, 26, 31, 37, 38]			Raven's Advanced Progressive Matrix (+)[10, 39]
		Matrix Reasoning Test (+)[37]		Wechsler Adult Intelligence Scale Logical Memory(+)[10, 18]	Zoo Map Test (+)[15, 40]
		Frontal Assessment Battery (+) [4, 10, 41]		Scenery Picture Memory Test (+)[14, 42]	0 and 2 Back Accuracy (+)[10]

	Wechsler Adult Intelligence Scale Digit Span Backward(+)[22]	Pictorial Memory Test-2(+)[29, 43]	Floor Maze Test(-)[44, 45]
	Verbal Fluency (Category Fluency in Animals)(+)[22]	Digit Span Test (+)[35]	Mental Rotation Test (+)[15, 46]
	BETA III General Non-Verbal Intelligence Test-BETA II Matrix Reasoning(+)[29, 32]		Chang Gung University Orthographic al Fluency Test (+)[4, 47]
	Digit Symbol Substitution Test(+)[35]		Tower of Hanoi Test (-)[6, 48]
			the 16-item Free and Cued Recall Task (+)(Letter and Category Fluency)[6, 49]

The supplementary table lists the cognitive tests that were included in the meta-analysis and, based on indications reported in the included studies, the cognitive domains assessed by each test. Domains were selected based on the study of Stuckenschneider and colleagues [50] A (+) indicates a positive direction of the scores (higher score, better cognitive performance), while a (-) indicates a negative direction of the score (lower score, better cognitive performance).

1. Folstein, M.F., S.E. Folstein, and P.R. McHugh, *“Mini-mental state”: A practical method for grading the cognitive state of patients for the clinician.* Journal of Psychiatric Research, 1975. **12**(3): p. 189-198.https://doi.org/10.1016/0022-3956(75)90026-6
2. Ansai, J.H., et al., *Effects of the Addition of a Dual Task to a Supervised Physical Exercise Program on Older Adults' Cognitive Performance.* Journal of Aging and Physical Activity, 2017. **25**(2): p. 234-239.10.1123/japa.2016-0094
3. Horata, E.T., S.Y. Cetin, and S. Erel, *Effects of individual progressive single- and dual-task training on gait and cognition among older healthy adults: a randomized-controlled comparison study.* European Geriatric Medicine, 2021. **12**(2): p. 363-370.10.1007/s41999-020-00429-5
4. Chen, Y.L., et al., *Dual-task multicomponent exercise-cognitive intervention improved cognitive function and functional fitness in older adults.* Aging Clinical and Experimental Research, 2023. **35**(9): p. 1855-1863.10.1007/s40520-023-02481-0
5. Uysal, I., et al., *Aerobic exercise and dual-task training combination is the best combination for improving cognitive status, mobility and physical performance in older adults with mild cognitive impairment.* Aging Clinical and Experimental Research, 2023. **35**(2): p. 271-281.10.1007/s40520-022-02321-7

6. Ayed, I.B., et al., *Mid-Term and Long-Lasting Psycho-Cognitive Benefits of Bidomain Training Intervention in Elderly Individuals with Mild Cognitive Impairment.* European Journal of Investigation in Health Psychology and Education, 2024. **14**(2): p. 284-298.10.3390/ejihpe14020019
7. Baek, J.E., et al., *Effects of dual-task resistance exercise on cognition, mood, depression, functional fitness, and activities of daily living in older adults with cognitive impairment: a single-blinded, randomized controlled trial.* Bmc Geriatrics, 2024. **24**(1).10.1186/s12877-024-04942-1
8. Han, C.L., et al., *Aerobic exercise combined with varied duration cognitive training to improve age-related cognitive impairment: A randomized controlled trial.* Geriatric Nursing, 2026. **70**.10.1016/j.gerinurse.2026.103981
9. Hatta, T., et al., *RELIABILITY AND VALIDITY OF THE DIGIT CANCELLATION TEST, A BRIEF SCREEN OF ATTENTION.* PSYCHOLOGIA, 2012. **55**(4): p. 246-256.10.2117/psysoc.2012.246
10. Takeuchi, H., et al., *Effects of Simultaneously Performed Dual-Task Training with Aerobic Exercise and Working Memory Training on Cognitive Functions and Neural Systems in the Elderly.* Neural Plasticity, 2020. **2020**.10.1155/2020/3859824
11. Barnes, D.E., et al., *The mental activity and exercise (MAX) trial: A randomized controlled trial to enhance cognitive function in older adults.* JAMA Internal Medicine, 2013. **173**(9): p. 797-804.10.1001/jamainternmed.2013.189
12. Reitan, R.M., *The relation of the trail making test to organic brain damage.* J Consult Psychol, 1955. **19**(5): p. 393-4.10.1037/h0044509
13. Rezola-Pardo, C., et al., *Comparison between multicomponent and simultaneous dual-task exercise interventions in long-term nursing home residents: the Ageing-ONDUAL-TASK randomized controlled study.* Age and Ageing, 2019. **48**(6): p. 817-823.10.1093/ageing/afz105
14. Castaño, L.A.A., et al., *Resistance Training Combined With Cognitive Training Increases Brain Derived Neurotrophic Factor and Improves Cognitive Function in Healthy Older Adults.* Front Psychol, 2022. **13**: p. 870561.10.3389/fpsyg.2022.870561
15. Béraud-Peigné, N., P. Maillot, and A. Perrot, *The effects of a new immersive multidomain training on cognitive, dual-task and physical functions in older adults.* GeroScience, 2023.10.1007/s11357-023-00952-w
16. Ismail, N.A. and H.A. Yusof, *The effect of brain-derived neurotrophic factor (BDNF) Val66Met gene polymorphism on cognitive performance of aging brain in response to an 8-week of exergames training.* Sport Sciences for Health, 2025. **21**(1): p. 363-378.10.1007/s11332-024-01267-9
17. Meekum, S., et al., *Enhancing Global Cognition and Executive Functions Through Water-Based Exercise in Mild Cognitive Impairment: A Randomized Controlled Trial.* Life-Basel, 2025. **15**(3): p. 13.10.3390/life15030420
18. Wechsler, D., *MEM-III, Échelle clinique de mémoire de Wechsler: manuel*. 2001: les Éd. du Centre de psychologie appliquée.
19. Fabre, C., et al., *Improvement of cognitive function by mental and/or individualized aerobic training in healthy elderly subjects.* Int J Sports Med, 2002. **23**(6): p. 415-21.10.1055/s-2002-33735
20. Ball, K. and C. Owsley, *The useful field of view test: a new technique for evaluating age-related declines in visual function.* J Am Optom Assoc, 1993. **64**(1): p. 71-9
21. Smith, T., N. Gildeh, and C. Holmes, *The Montreal Cognitive Assessment: Validity and Utility in a Memory Clinic Setting.* The Canadian Journal of Psychiatry, 2007. **52**(5): p. 329-332.10.1177/070674370705200508

22. Li, F., et al., *A cognitively enhanced online Tai Ji Quan training intervention for community-dwelling older adults with mild cognitive impairment: A feasibility trial.* BMC Geriatrics, 2022. **22**(1).10.1186/s12877-021-02747-0
23. Silva, A.F., et al., *The Effect of Aerobic or Strength Training in Elderly with Cognitive Decline: The Fit4Alz Project.* Journal of Sports Science and Medicine, 2025. **24**(1): p. 172-186.10.52082/jssm.2025.172
24. de Almeida, M.L., et al., *Cognitive Training Combined with Multicomponent Exercise Improves Biopsychosocial Outcomes in Healthy Older Adults: a Randomized Trial.* Journal of Cognitive Enhancement, 2026. **10**(1): p. 65-80.10.1007/s41465-026-00347-7
25. Eriksen, B.A. and C.W. Eriksen, *Effects of noise letters upon the identification of a target letter in a nonsearch task.* Perception & Psychophysics, 1974. **16**(1): p. 143-149.10.3758/BF03203267
26. Damirchi, A., F. Hosseini, and P. Babaei, *Mental Training Enhances Cognitive Function and BDNF More Than Either Physical or Combined Training in Elderly Women With MCI: A Small-Scale Study.* American Journal of Alzheimers Disease and Other Dementias, 2018. **33**(1): p. 20-29.10.1177/1533317517727068
27. Schmidt, M., *Rey Auditory Verbal Learning Test: RAVLT : a Handbook*. 1996: Western Psychological Services.
28. Sunderland, T., et al., *Clock Drawing in Alzheimer's Disease.* Journal of the American Geriatrics Society, 1989. **37**(8): p. 725-729.https://doi.org/10.1111/j.1532-5415.1989.tb02233.x
29. Biehl-Printes, C., et al., *Unraveling the unparalleled benefits of orienteering versus hiking on gait performance and cognition: A randomized clinical trial.* Archives of Gerontology and Geriatrics, 2024. **117**.10.1016/j.archger.2023.105201
30. Cambraia, S., *Teste de Atenção Concentrada (AC). Manual.* São Paulo: Vetor, 2003
31. Anderson-Hanley, C., et al., *The Aerobic and Cognitive Exercise Study (ACES) for Community-Dwelling Older Adults With or At-Risk for Mild Cognitive Impairment (MCI): Neuropsychological, Neurobiological and Neuroimaging Outcomes of a Randomized Clinical Trial.* Frontiers in Aging Neuroscience, 2018. **10**.10.3389/fnagi.2018.00076
32. Rabelo, I., et al., *Teste não verbal de inteligência geral–BETA III-Subtestes raciocínio matricial e códigos.* Manual Técnico. São Paulo: Casa do Psicólogo, 2011
33. Harrison, J., et al., *A Neuropsychological Test Battery for Use in Alzheimer Disease Clinical Trials.* Archives of Neurology, 2007. **64**(9): p. 1323-1329.10.1001/archneur.64.9.1323
34. Troyer, A.K., M. Moscovitch, and G. Winocur, *Clustering and switching as two components of verbal fluency: Evidence from younger and older healthy adults.* Neuropsychology, 1997. **11**(1): p. 138-146.10.1037/0894-4105.11.1.138
35. Wang, Y.X., et al., *Comparative effects of cognitive and instability resistance training versus instability resistance training on balance and cognition in elderly women.* Scientific Reports, 2024. **14**(1).10.1038/s41598-024-77536-x
36. Van der Elst, W., et al., *The Stroop Color-Word Test:Influence of Age, Sex, and Education; and Normative Data for a Large Sample Across the Adult Age Range.* Assessment, 2006. **13**(1): p. 62-79.10.1177/1073191105283427
37. Combourieu Donnezan, L., et al., *Effects of simultaneous aerobic and cognitive training on executive functions, cardiovascular fitness and functional abilities in older adults with mild cognitive impairment.* Mental Health and Physical Activity, 2018. **15**: p. 78-87.10.1016/j.mhpa.2018.06.001
38. Strauss, E., E.M.S. Sherman, and O. Spreen, *A Compendium of Neuropsychological Tests: Administration, Norms, and Commentary*. 2006: Oxford University Press.
39. Raven, J., J.C. Raven, and J.H. Court, *Manual for Raven's Progressive Matrices and Vocabulary Scales*. 1998: Oxford Psychologists Press.

40. Oosterman, J.M., M. Wijers, and R.P.C. Kessels, *Planning or Something Else? Examining Neuropsychological Predictors of Zoo Map Performance.* Applied Neuropsychology: Adult, 2013. **20**(2): p. 103-109.10.1080/09084282.2012.670150
41. Dubois, B., et al., *The FAB: a Frontal Assessment Battery at bedside.* Neurology, 2000. **55**(11): p. 1621-6.10.1212/wnl.55.11.1621
42. Takechi, H. and H.H. Dodge, *Scenery Picture Memory Test: A new type of quick and effective screening test to detect early stage Alzheimer's disease patients.* Geriatrics & Gerontology International, 2010. **10**(2): p. 183-190.https://doi.org/10.1111/j.1447-0594.2009.00576.x
43. Rueda, F.J.M. and F.F. Sisto, *Teste pictórico de memória (TEPIC-M).* São Paulo: Vetor, 2007
44. Porteus, S.D., *Porteus Maze Tests: Fifty years' application.* 1965
45. Oliveira, L.M., et al., *2D Virtual Reality-Based Exercise Improves Spatial Navigation in Institutionalized Non-robust Older Persons: A Preliminary Data Report of a Single-Blind, Randomized, and Controlled Study.* Frontiers in Neurology, 2021. **11**.10.3389/fneur.2020.609988
46. Vandenberg, S.G. and A.R. Kuse, *Mental Rotations, a Group Test of Three-Dimensional Spatial Visualization.* Perceptual and Motor Skills, 1978. **47**(2): p. 599-604.10.2466/pms.1978.47.2.599
47. Hung, Y.-H., T.-L. Wang, and C.-C. Yang, *Chinese character fluency test: an investigation of psychometric properties.* The Clinical Neuropsychologist, 2016. **30**(sup1): p. 1429-1442.10.1080/13854046.2016.1166266
48. Anderson, J.R. and S. Douglass, *Tower of Hanoi: evidence for the cost of goal retrieval.* J Exp Psychol Learn Mem Cogn, 2001. **27**(6): p. 1331-46
49. Dion, M., et al., *Normative data for the Rappel libre/Rappel indicé à 16 items (16-item Free and Cued Recall) in the elderly Quebec-French population.* Clin Neuropsychol, 2015. **28 Suppl 1**: p. S1-19.10.1080/13854046.2014.915058
50. Stuckenschneider, T., et al., *The Effect of Different Exercise Modes on Domain-Specific Cognitive Function in Patients Suffering from Parkinson's Disease: A Systematic Review of Randomized Controlled Trials.* Journal of Parkinson's Disease, 2019. **9**(1): p. 73-95.10.3233/jpd-181484
